# Bioengineering approaches to trained immunity: Physiologic targets and therapeutic strategies

**DOI:** 10.7554/eLife.106339

**Published:** 2025-07-23

**Authors:** Hannah Riley Knight, Marie Kim, Nisha Kannan, Hannah Taylor, Hailey Main, Emily Azcue, Aaron Esser-Kahn

**Affiliations:** 1 Pritzker School of Molecular Engineering, University of Chicago Chicago United States; 2 Biological Sciences Division, University of Chicago Chicago United States; 3 Department of Chemistry, University of Chicago Chicago United States; Radboud University Nijmegen Medical Centre Netherlands; Indian Institute of Science Education and Research (IISER) India

**Keywords:** bioengineering, trained immunity, nanotherapeutics, biomechanics, cellular engineering, machine learning

## Abstract

Trained immunity presents a unique target for modulating the immune response against infectious and non-infectious threats to human health. To address the unmet need for training-targeted therapies, we explore bioengineering methods to answer research questions and address clinical applications. Current challenges in trained immunity include self-propagating autoinflammatory disease, a lack of controllable cell and tissue specificity, and the unintentional induction of training by known drugs and diseases. The bioengineering tools discussed in this review (nanotherapeutics, biomechanical modulation, cellular engineering, and machine learning) could address these challenges by providing additional avenues to modulate and interrogate trained immunity. The preferential activation of peripheral or central training has not yet been achieved and could be accessed using nanoparticle systems. Targeted delivery of training stimuli using nanocarriers can enrich the response in various cell and organ systems, while also selectively activating peripheral training in the local tissues or central trained immunity in bone marrow progenitor cells. Beyond chemical- or pathogen-based activation of training, force-based cues, such as interaction with mechanoreceptors, can induce trained phenotypes in many cell types. Mechanotransduction influences immune cell activation, motility, and morphology and could be harnessed as a tool to modulate training states in next-generation therapies. For known genetic and epigenetic mediators of trained immunity, cellular engineering could precisely activate or deactivate programs of training. Genetic engineering could be particularly useful in generating trained cell-based therapies like chimeric antigen receptor (CAR) macrophages. Finally, machine learning models, which are rapidly transforming biomedical research, can be employed to identify signatures of trained immunity in pre-existing datasets. They can also predict protein targets for previously identified inducers of trained immunity by modeling drug-protein or protein-protein interactions in silico. By harnessing the modular techniques of bioengineering for applications in trained immunity, training-based therapies can be more efficiently translated into clinical practice.

## Introduction

Bioengineering is a complex and often nebulous concept encompassing many overlapping scientific disciplines (Nature, 2023). Fundamentally, bioengineering is the application of engineering concepts to biological systems, either for the generation of a product or the enhancement of the biological system *as* the product (Ideker et al., 2006; Citron and Nerem, 2004). The earliest mentions of bio- or biomedical engineering in scientific literature appeared toward the end of the Second World War, but it would not join departments of engineering schools in earnest until the 1970s ([Bibr bib76][Bibr bib137]). By then, developments in medical diagnostics, imaging, and molecular biology allowed for the precise study and manipulation of biological systems required for such a ‘systems engineering’ approach. Scientists had the tools to introduce precise, well-controlled perturbations and measure their effects. This shift led to the practical application of bioengineering: the development of tools, technologies, delivery systems, and measurement methods to understand and manipulate biology. Today, these experimental approaches range from quantum-based biosensors to genome editing to patient-derived organoids (Kalkal et al., 2020; Cox et al., 2015; Too et al., 2021).

Bioengineering is not limited to experimental techniques in research labs. It has also found its way into clinical practice, with the first nanoparticle-based therapeutic, Doxil, gaining FDA approval in 1995 (Barenholz, 2012). Cell and gene therapies, which were once considered science fiction are now widely accepted, including autologous stem cell therapies for a variety of conditions, chimeric antigen receptor T cells (CAR-Ts) for blood cancers, and gene therapies for single-gene disorders such as sickle cell disease (Leonard and Tisdale, 2024; Larson and Maus, 2021; Wang et al., 2021; Lee et al., 2024; Zhang and Cheng, 2023). Despite the massive organizational and financial investments in bioengineering, too little attention has been paid to the innate immune system. Innate immune cells were considered primordial, only responding to evolutionarily conserved patterns and lacking the ability to learn from their experiences. This perception changed with the introduction of trained immunity by Mihai Netea and colleagues in 2011 (Netea et al., 2011).

Trained immunity is defined as the epigenetic and metabolic reprogramming of innate immune cells. Until recently, inducers of trained immunity have been limited to endogenous signaling molecules and pathogen-derived sources that directly activate pattern recognition receptors (PRRs) such as toll-like receptors (TLRs). However, non-inflammatory drugs and drug-like compounds identified by high-throughput screening induce training in vitro and in vivo (Knight et al., 2024; Ajit et al., 2024). This expansion of known inducers of trained immunity offers alternative methods to induce training without initial immune activation. In addition, new work has further suggested that training is not a universal state but is rather stimulus-specific, resulting in different effector responses determined by initial activation (O’Farrell et al., 2025). Together, these new insights highlight the importance of tailoring training based on the desired outcome.

**Figure 1.**
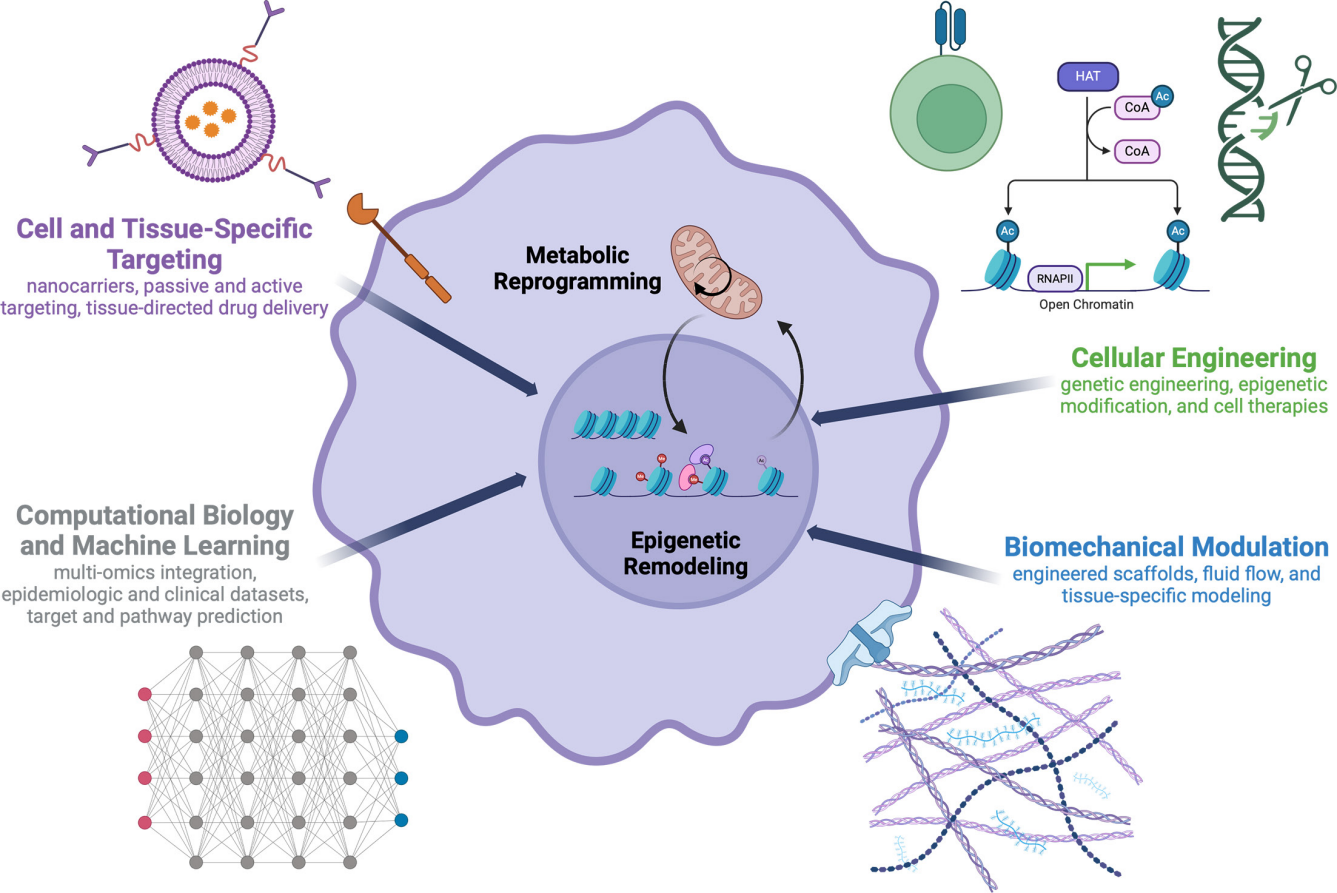
Proposed bioengineering approaches to understanding and applying trained immunity. This figure was created with BioRender.com.

Our motivation for this article is twofold. First, we seek to introduce bioengineering techniques and perspectives to those studying the mechanistic biology of trained immunity. Second, we aim to promote trained immunity as a therapeutic target among bioengineers. Applying bioengineering approaches, including targeted delivery, biomechanical modulation, genetic engineering, and machine learning, can further expand the repertoire of trained immunity inducers and extend the reach of its clinical applications (Figure 1; Mulder et al., 2019).

Here, we explore the current state of bioengineered trained immunity and suggest avenues for future research and innovation. We will begin by defining central and peripheral training and outlining the key challenges in activating one or more modes of training. Then, we explore passive and active targeting approaches, including nanotherapeutics, as a potential solution. Next, mechanotransduction and cellular engineering approaches are discussed as both research and therapeutic methods. Finally, we propose the use of machine learning to identify new drug and protein targets and extract training-like signatures from preexisting clinical and epidemiologic datasets. We hope to encourage the translation of trained immunity from bench to bedside through the application of bioengineering techniques.

### Central vs. peripheral trained immunity

Central trained immunity is the epigenetic and metabolic reprogramming of the long-lived progenitor cells of the bone marrow, hematopoietic stem cells (HSCs) (Kaufmann et al., 2018). Peripheral trained immunity is the immunometabolic reprogramming of mature, terminally differentiated, tissue-resident cells such as alveolar macrophages and skin epithelial cells (Geller et al., 2022). While there is interest in inducing central and peripheral trained immunity either independently or in combination, the challenge of disentangling these two types of training has not yet been overcome. Furthermore, without the ability to selectively activate one type of training or the other, it is difficult to determine the contribution of each type of training to the immune response.

#### Maladaptive central training in hematopoietic stem cells: a target for training suppression

Depending on the original stimulus, training has been observed to endure as long as one year (Netea et al., 2020). Induction of central training in HSCs occurs in the bone marrow and allows for epigenetic reprogramming, which is passed down to daughter cells that eventually differentiate into trained effector cells (e.g. monocytes and macrophages) (Netea et al., 2016). The mechanisms by which HSCs in the bone marrow niche acquire trained immunity is being extensively investigated, with many insights stemming from studies using systemic delivery of Bacille Calmette-Guérin (BCG) and β-glucan (Kaufmann et al., 2018; de Laval et al., 2020; Mills et al., 2024; Moorlag et al., 2020).

Both β-glucan and BCG reprogram myeloid progenitors in the bone marrow (Chavakis et al., 2019). In mice, central training by β-glucan is driven by perturbations in glycolysis, cholesterol biosynthesis, NLRP3 inflammasome activation, and IL-1β or granulocyte-macrophage colony-stimulating factor (GM-CSF) signaling (Mitroulis et al., 2018; Kalafati et al., 2020). In systemic BCG vaccination, Kaufmann et al., 2018 have demonstrated reprogramming of HSCs towards myelopoiesis, which leads to protective trained immunity in a mouse model of tuberculosis (Kaufmann et al., 2018). The memory-like function of HSCs can explain how short-lived immune cells such as monocytes are imprinted with durable epigenetic marks.

Persistent activation of immune cells from central trained immunity may drive pathology of chronic inflammatory diseases such as diabetes, atherosclerosis, and myocardial infarction (Mills et al., 2024; Edgar et al., 2021; van der Heijden et al., 2020; Dong et al., 2024). Overactive immune responses can damage healthy tissues and worsen disease progression, while also increasing susceptibility to other diseases, including cancer (Koelwyn et al., 2020).

The NLRP3 inflammasome pathway has been implicated in trained immunity and in various autoinflammatory disorders, which must be considered in the development of training-based therapeutics (Lee et al., 2023a; Li et al., 2022; Christ et al., 2018). For example, in a mouse model of periodontitis, an oral inflammatory disease, increased systemic inflammation led to IL-1 receptor-mediated maladaptive central training of HSCs. These mice were then more susceptible to experimental arthritis (Li et al., 2022). This finding suggests that acquiring central training in one autoinflammatory condition increases the risk of developing other training-driven comorbidities.

Maladaptive training of the bone marrow can also be induced by highly processed, western diets (Christ et al., 2018). Altered immune metabolism from central training is linked to metabolic syndromes and type 2 diabetes through enhanced production of inflammatory cytokines (Choudhury et al., 2021). These NLRP3 inflammasome-mediated trained immunity pathways have also been observed in humans in diabetes and atherosclerosis (Edgar et al., 2021; Bekkering et al., 2016). In one study, bone marrow was transplanted from diabetic mice to mice with normal glycemic index; these otherwise healthy mice became prone to atherosclerosis and developed lesions with necrotic, lipid-rich cores. This phenotype mirrors the increased rates of atherosclerosis observed in diabetic humans (Choudhury et al., 2004).

Beyond chronic conditions and environmental factors, central training and its consequences can also occur following acute trauma. For example, a study with experimental myocardial infarction (MI) showed induction of maladaptive training of myeloid progenitors, and the trained phenotype could be transferred by bone marrow transplantation to apolipoprotein-E deficient mice on a high-fat diet (Dong et al., 2024). When compared with controls that received untrained bone marrow, the mice that received bone marrow from post-MI mice exhibited elevated levels of systemic IL-1β and TNF-ɑ and increased atherosclerotic lesions.

In each of these experimental models and patient cohorts, training drives susceptibility to secondary inflammatory conditions. These direct relationships between central training and autoinflammatory diseases give pause when considering central trained immunity as a therapeutic target. If training-based therapeutics access the bone marrow and induce central training, then there is a risk of inducing or worsening these training-mediated autoinflammatory conditions. However, strategies to directly inhibit central trained immunity may reduce hyper-inflammation in chronic diseases while preserving peripheral immune responses for local defense, providing a new therapeutic target for these common chronic diseases. In each of these diseases, maladaptive central training is implicated as a direct driver of pathology, and pathology can be reproduced by the adoptive transfer of the trained HSCs into naïve mice. Therefore, selective suppression of training in HSCs via bioengineering approaches such as nanotherapeutics, gene therapies, or epigenetic-directed inhibitors are promising strategies to suppress the progression of these autoinflammatory diseases. Such methods will be discussed further in the *Cellular Engineering* section.

#### Peripheral trained immunity in immune and non-immune cells: targets for tissue-specific protection

**Figure 2.**
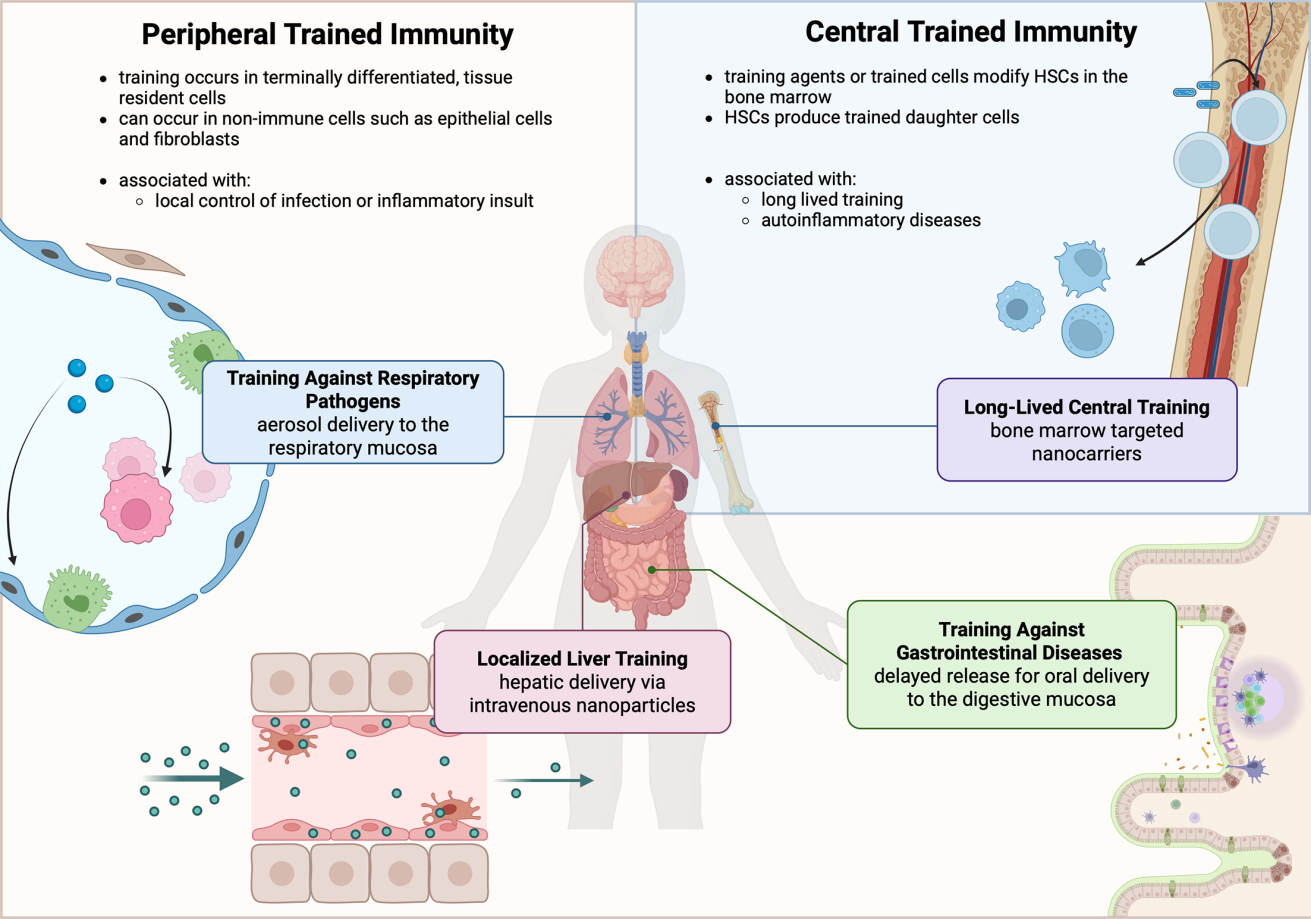
Characteristics of central and peripheral trained immunity and target tissues for therapeutic induction of trained immunity. (Top Right) Central training occurs in progenitor cells of the bone marrow (shown here via Bacille Calmette-Guérin, BCG training of hematopoietic stem cells, HSCs), leading to long-lived, multi-generational training in daughter cells. Central training is also directly implicated in the pathogenesis of autoinflammatory diseases, such as atherosclerosis and diabetes. Directed targeting to the bone marrow can access hematopoietic stem cells for long-lived central training. (Left and Bottom) Peripheral trained immunity can encompass tissue-resident innate immune cells and stromal cells, such as epithelial cells and fibroblasts (shown here with small molecule training in the alveoli of the lung, Kupffer cells in the liver, and Peyer’s Patches in the small intestine). Peripheral training can provide local resistance to infection, cancer, and other inflammatory insults. A combination of passive and active targeting approaches can be used to access peripheral training in the lung, gut, and liver. Respiratory delivery can be achieved with aerosols, gastrointestinal delivery can be targeted with delayed release systems, and hepatic delivery can be achieved with intravenous delivery of nanoparticles, which naturally accumulate in the liver. This figure was created with BioRender.com.

Peripheral trained immunity is local and can be more transient compared to central trained immunity (Figure 2). This form of immunity is induced by direct environmental interactions with pathogens or other stimuli in tissue-resident cells, in tissues such as the skin and lungs (Netea and Joosten, 2018). A study by Yao et al., 2018 found that the formation and maintenance of alveolar macrophages show memory features that occur independently of monocytes or bone marrow progenitors using a model of adenovirus lung infection (Yao et al., 2018). Other tissue-resident macrophages, such as Kupffer cells or microglia, self-renew independently of HSCs, making them promising targets for lasting innate training (Liu et al., 2022; Heng et al., 2021; Gomez Perdiguero et al., 2015). In an immunosuppressive tumor microenvironment, β-glucan-mediated trained innate immune responses establish an anti-tumor microenvironment that may be sufficient to eradicate the typically immunosuppressive pancreatic tumor, further cementing the effectiveness of peripheral trained immunity in combating disease (Geller et al., 2022). The same group demonstrated the role of interstitial macrophages in the host resistance of metastatic lesions in an intravenous melanoma model (Ding et al., 2023).

Like innate immune cells, many other cell types are also capable of peripheral training with both pathogenic consequences and therapeutic benefits. Stromal cells can exhibit epigenetic and metabolic changes in the local tissue environment, consistent with peripheral training. For example, epithelial cells express PRRs, like immune cells, and produce immunomodulators and antimicrobial compounds when exposed to pathogens (Schaefer et al., 2004; Naik et al., 2017). One study observed that skin epithelial stem cells exhibit features consistent with innate immune memory. Using imiquimod in a model of skin inflammation, the authors observed that the skin previously exposed to imiquimod not only reacted more quickly to an unrelated secondary challenge but also exhibited more rapid wound healing than skin of naïve mice (Naik et al., 2017). This memory response has been attributed to epigenetic changes in the stem cells of the skin.

Inflammatory memory in epithelial cells is not limited to the skin—it has also been reported in the gut. Intestinal stromal cells also respond to bacterial pathogens and have been suggested to exhibit immunological memory (Novakovic and Stunnenberg, 2017; Owens, 2015). Intestinal stromal cells produce prolonged proinflammatory cytokines that aid in recruiting immune cells to the site of infection during a secondary challenge (Owens, 2015; Owens and Simmons, 2013; Owens et al., 2013). Further studies reported that fibroblasts also respond to pathogens by interacting with other cells through the production of inflammatory signals (Kaufman et al., 2001). Studies have revealed that fibroblasts are involved in the persistence of inflammation: when activated during infection, tissue repair triggers a protective immune response through the recruitment of immune cells to clear infections and rebuild tissue (Kaufman et al., 2001; Bautista-Hernández et al., 2017; Flavell et al., 2008).

Together, these studies highlight the importance of tissue-specific training cues and their ability to halt the spread of infection. The variable durability, cell specificity, and tissue-specific response of peripheral training provide opportunities to use bioengineering to create tissue-specific vaccines and therapies that enhance host defenses while minimizing the risk of harmful inflammatory responses posed by central trained immunity. Approaches to selectively target peripheral trained immunity will be explored in the *Passive and Active Targeting Approaches*.

### Therapeutic targets and challenges for trained immunity

Selectively modulating peripheral innate immune memory could provide opportunities to create therapies that strengthen host defense while minimizing harmful inflammatory responses, albeit with many challenges. For example, local tissue training can, in some cases, lead to systemic therapeutic effects. Talimogene laherparepvec (T-VEC), a modified herpes simplex virus engineered to encode GM-CSF, exerts anti-tumor effects through direct oncolysis at the site of administration but also through innate immune cell-driven systemic responses. This virus has been approved by the FDA to treat unresectable melanoma (Kaufman et al., 2022; Jennings et al., 2019). Another example where trained immunity has been attributed to increased systemic effects includes the anti-tumor response following intratumoral administration of mRNA encoding IL-12, IFN-ɑ, IL-15, and GM-CSF (Hotz et al., 2021). Delivering mRNA encoding tumor-specific antigens or cytokines to antigen-presenting cells (APCs) can result in a systemic anti-tumor response mediated by the local innate immune activation (Rojas et al., 2023; Cafri et al., 2020; Sahin et al., 2020). Most recently, trained immunity has been applied to sepsis treatment to overcome immune paralysis using the potential of apolipoprotein nanotechnology (Schrijver et al., 2023. Schrijver et al., 2023) discovered that apolipoprotein nanoparticles loaded with IL-4 could control sepsis via induction of trained immunity in myeloid cells (Schrijver et al., 2023). In each of these models, the training of peripheral innate immune cells coordinated a protective systemic response.

Exploiting trained immunity mechanisms could also enhance vaccine efficacy, particularly for populations with weakened immune systems – notably the elderly, young children, and the immunocompromised. Trained memory has been attributed to the nonspecific effects of vaccines against unrelated illnesses which have been demonstrated in many pre-clinical and clinical studies (Ziogas and Netea, 2022; Del Fresno et al., 2021; Goodridge et al., 2016; Benn et al., 2013; Higgins et al., 2016). Animal studies demonstrated that BCG vaccination conferred protection against candidemia in severe combined immunodeficiency disease (SCID) mice, which lack all adaptive immune cells, alluding to the protective effects of innate memory (Kleinnijenhuis et al., 2012). It has also been observed that BCG-induced training conferred nonspecific protection against malaria and viral infection in humans (Arts et al., 2018; Walk et al., 2019). In elderly patients, non-specific BCG protection has been observed against respiratory infections (Giamarellos-Bourboulis et al., 2020). Similarly, a new tuberculosis vaccine, VPM1002, also conferred non-specific protection against severe respiratory infections in the elderly (Blossey et al., 2023). Another example of the broader protection offered by trained immunity is MTBVAC, a live attenuated tuberculosis vaccine that confers heterologous protection against *Streptococcus pneumoniae* (Tarancón et al., 2020). Signatures of trained innate immunity were also observed in humans after SARS-CoV-2 infection. The Pfizer mRNA vaccine, BNT162b2, induced short-term epigenetic memory in innate immune cells (Yamaguchi et al., 2022).

Vaccine adjuvants have also been demonstrated to induce trained immunity signatures. For example, the addition of oil-based adjuvant AS03 in an influenza vaccine induces epigenetic changes in myeloid cells, generating in vitro resistance to unrelated viruses such as Dengue and Zika (Wimmers et al., 2021). A TLR7/8 agonist, 3M052, also drives epigenetic and transcriptomic modifications in murine myeloid cells (Lee et al., 2022). Thus, trained immunity-related epigenetic changes in myeloid cells may increase the responsiveness of these trained cells to subsequent vaccine doses and improve the crosstalk between lymphocytes and myeloid cells during vaccination. In clinical applications, trained immunity can independently enhance protection against diseases and amplify the response to immune-targeted therapies, like vaccines and cancer treatments.

### Passive and active targeting approaches

#### Cellular delivery

While both central and peripheral training can play a role in therapeutic protection and pathogenesis, targeting these compartments directly within cells and tissues remains an unaddressed challenge. One approach to cell-specific, targeted delivery of trained immunity-inducing stimuli is nanocarriers. Nanocarriers can target many tissues, including the gut and lung, and can be directed to nearly any cell type. However, they are naturally suited to target cells of the mononuclear phagocyte system, especially macrophages, which are primary drivers of trained immunity.

Targeting specific cell types via surface markers is a common bioengineering strategy in vaccines, treatment of cancer, and other inflammatory diseases (Ellipilli et al., 2023; Hua et al., 2015; Shi et al., 2024; Kim et al., 2013). Conjugated or self-assembled delivery systems can be decorated with natural ligands, synthetic peptides, or nanobodies with target-specific binding affinity as a method of active targeting (Arias et al., 2015; Zhou et al., 2025; Lee et al., 2023b; Tian et al., 2022; Tong et al., 2021). Depending on the designs of these targeting moieties and the function of their cognate receptor, the binding event can induce drug uptake, activation of signaling, or inhibition of signaling (Bajracharya et al., 2022; Slezak et al., 2022; Duan and Luo, 2021). Encapsulation in nanocarriers may also reduce off-target effects and toxicities that limit the dosing of a drug, as in the case of the chemotherapeutic doxorubicin when formatted in liposomes as Doxil (Waterhouse et al., 2001).

In addition to active targeting approaches, nanoparticle morphology can be designed to influence uptake in specific tissues or cell types via passive targeting. Inherent characteristics of nanomaterials, including surface charge, shape, size, and topography are known to affect their uptake and distribution in vivo (Mitragotri and Lahann, 2009; Champion and Mitragotri, 2006; Augustine et al., 2020). For example, to bias delivery to phagocytic cells, such as macrophages, size- and morphology-restricted nanoparticles can be useful. Macrophages preferentially uptake particles upwards of 400 nm in size, whereas neutrophils prefer elongated, rod-shaped particles (Safari et al., 2020; Li et al., 2021). These nanoparticles can have many different compositions and characteristics, from complex lipid nanoparticles to self-assembling polymersomes, each with different properties and benefits (Table 1). Liposome-based systems, in particular, offer a non-inflammatory, inexpensive method for the delivery of both hydrophobic and hydrophilic cargo (Minocha and Kumar, 2022).

**Table 1.** Some nanocarrier types and characteristics.

Type	Advantages	Disadvantages
Polymeric Lu et al., 2021	Stability More amenable to surface decoration Controlled release Can be designed for stimulus-specific degradation	No endosomal escape mechanism Lower loading ability Immunogenic Difficult to synthesize in a controlled fashion
Liposomes, Micelles, and Emulsions Lu et al., 2021	Modular components FDA-approved products Biocompatible Simple to produce Can load hydrophobic and hydrophilic cargo	Scale up Extra-hepatic delivery is challenging Less stable
Lipid Nanoparticles Lu et al., 2021	FDA-approved products Ideal for nucleic acid cargo Endosomal escape Biocompatible	Immunogenic Extra-hepatic delivery is challenging Less stable, especially at 4 °C
Lipoprotein Damiano et al., 2013; Thaxton et al., 2016	Biocompatible Biomimicry Natural metabolic trafficking target	Complex - quality control and scale up is costly Purification challenges
Exosomes Colombo et al., 2014	Physiologic delivery system Low risk of immunogenicity Longer circulation times	Heterogeneous loading Labor-intensive isolation

Several groups are already pursuing various nanocarrier-based delivery systems to induce trained immunity. Exosomes, either engineered ex vivo or endogenously derived, can hijack the native intracellular communication system to induce or modulate training, as demonstrated via both bacterial and stem cell sources (Liu et al., 2024b; Feng et al., 2020). In one study from Mulder et al., 2019, muramyl dipeptide (MDP) was lipidated and loaded onto a scaffold of apolipoprotein A (ApoA), the primary component of high-density lipoprotein (HDL) (Priem et al., 2020). This biomimetic nanocarrier induced trained immunity in mice and significantly improved their response to checkpoint inhibition in a B16F10 melanoma model. In an alternative approach, β-glucan was encapsulated in poly lactic-co-glycolic acid (PLGA) nanoparticles, resulting in extended release of β-glucan and prolongation of the training period (Ajit et al., 2022). Mice that were trained with the β-glucan PLGA nanoparticles significantly resisted engraftment of B16F10 tumors. Due to their inherent interactions with innate immune cells, particularly the cells that comprise the mononuclear phagocyte system, nanoparticles are an attractive delivery modality for inducing trained immunity in a more precise and controllable manner. While nanoparticle-based induction of trained immunity is being explored in preclinical studies, there are no ongoing clinical trials using this approach.

#### Tissue delivery

Similar methods can be used to target delivery to specific tissues rather than individual cell types, since nanocarriers also preferentially accumulate in different organs depending on their physical and chemical characteristics (Table 1, Figure 2; Wang et al., 2023b; Su et al., 2024). For example, intravenously administered nanoparticles naturally traffic to the liver and may be useful for inducing trained immunity to prophylactically combat septic liver injury or hepatitis (Kumar et al., 2023; Liu et al., 2024a; Wang et al., 2024; Hong et al., 2015). Intravesical delivery of BCG is currently used as a trained immunity-inducing treatment in bladder cancer; therefore, targeted delivery of other inducers of training to the kidneys and epithelial cells of the urogenital tract may offer a less invasive alternative (Alexandroff et al., 1999; Buffen et al., 2014). This strategy may also allow researchers to selectively induce peripheral or central trained immunity, depending on the goal of the treatment. Small molecules, in particular, can easily be formulated for topical delivery to the skin, aerosol-based delivery to the respiratory mucosa, or oral delivery to the gut with delayed release capabilities (Ma et al., 2021; Uhl et al., 2021; Souto et al., 2022; Wang et al., 2023a). Several groups have demonstrated that epithelial cells of mucosal surfaces are trained by inflammatory stimuli, which then alter the expression of key adhesion molecules on their surface (Subudhi et al., 2024; Russell et al., 2023; Rosenblum and Naik, 2022; Naik and Fuchs, 2022). This phenomenon presents an exciting opportunity to modulate susceptibility to environmental exposures, infectious or otherwise, by training mucosal surfaces directly. Nanoparticle systems may also be employed to reverse the effects of trained immunity in the case of maladaptive training. Therefore, there is much interest in employing nanoparticle-based systems for the precise delivery of trained immunity-modifying therapeutics.

### Biomechanical approaches

#### Mechanotransduction and epigenetics

A new, exciting approach to induce training is the manipulation of mechanical signaling. Mechanotransduction is critical to the ability of immune cells to communicate with adjacent or remote cells, to migrate to the site of infection, or to adapt to disrupted local environments. These processes rely on the cell’s ability to sense both the biochemical and mechanical changes around them (Wozniak et al., 2004; Geiger et al., 2009). Disruptions and changes in the stresses and strains in the extracellular matrix (ECM) can stem from numerous sources, such as degraded ECM during a bacterial infection. Cells sense such mechanical changes in their microenvironment via focal adhesions, a specialized complex of adhesion proteins. In particular, integrins are a superfamily of transmembrane cell receptors that comprise focal adhesions (Hood and Cheresh, 2002). Integrins are crucial mechanotransducers, converting mechanical forces from the ECM into intracellular biochemical signals and vice versa.

The activation of mechanical signaling stimulates intracellular signaling cascades that affect numerous cellular processes, such as differentiation, proliferation, tissue development, migration, or survival. The cross-linking of integrins and binding to their ligands activate downstream signaling proteins, which propagate the extracellular mechanical signals to the nucleus via the cytoskeletal network (Miranti and Brugge, 2002). Various nucleoskeletal machinery, namely the Linker of Nucleoskeleton and Cytoskeleton (LINC) complex, is then involved to propagate the signal through the nuclear envelope and nuclear lamina to reach the chromatin. Mechanical signals transduced to the nucleus can alter chromatin structure and activate various transcription factors (TFs) that drive specific regulation of gene expression. Studies showed that actomyosin contraction induced by a stiffer environment led to nuclear flattening, which caused nuclear localization and accumulation of Yes-associated protein (YAP) and transcriptional co-activator with PDZ-binding motif (TAZ) downstream. This cascade led to the strengthening of the focal adhesion and cytoskeletal network, as YAP/TAZ controls the activation of genes encoding focal adhesion proteins (Elosegui-Artola et al., 2017; Kim and Gumbiner, 2015; Nardone et al., 2017; Aragona et al., 2013).

Our current understanding of how mechanotransduction can alter and influence the transcriptomic and epigenetic landscape is still evolving. A recent study demonstrated that local stress applied to the cell surface led to chromatin stretching that depended on the loading direction (Tajik et al., 2016). Mechanical force-induced remodeling of chromatin also depends on the duration of force. Short durations of force applied to the cell surface, within seconds, initiate immediate stretching of the chromatin and upregulation of mechanosensitive genes, such as *Cav1* or *egr-1*, mediated by H3K9me3 demethylation (Sun et al., 2020). In contrast, longer exposure to force, in duration of hours, leads to dynamic adaptation by the chromatin to minimize the strain on the nucleus, an unconventional method to maintain genome integrity in response to mechanical deformation (Nava et al., 2020).

Interestingly, many studies note the importance of mechanical stimulation in the induction of longer-term changes in cell phenotypes by epigenetic modifications. For example, the lineage commitment of mesenchymal stem cells is reliant on mechanical cues. Mesenchymal stem cells (MSCs) seeded in stiffer substrates undergo osteogenic differentiation; in softer substrates, they undergo adipogenic or neuronal differentiation (Engler et al., 2006). Rapid ATP synthesis ensuing dynamic loading enables chromatin condensation, a key step in differentiation, showing the importance of mechanical loading on both metabolic and epigenetic regulation (Heo et al., 2016). Matrix stiffness can regulate nuclear translocation of both methyltransferases and acetyltransferases, inducing methylation, or chromatin condensation, and acetylation, or chromatin relaxation, respectively (Jang et al., 2021; Zhao et al., 2021; Song et al., 2024). In summary, mechanical force is a crucial cellular signal that controls gene expression and has potential as a robust method to manipulate epigenetics.

#### Scaffold-based manipulation of epigenetics

The forces applied onto cells by scaffolds or extracellular matrices provide stress-strain stimulation to cells often necessary for homeostasis. Consequently, changes in the scaffold can lead to altered cellular responses. For example, the high-tension environment made by the highly aligned and packed collagen fibers in tendons promotes an anabolic state in tenocytes at homeostasis. The loss of this tension leads to a decrease in chromatin accessibility in tenocytes, increasing the expression of a matrix of catabolic genes (Jones et al., 2023). In bone, sensitivity to fluid shear stress has been shown to be enhanced by Sirtuin-3, a histone deacetylase, which also plays an important role in regulating bone mass (Li et al., 2023a). In another study with chondrocytes, three-dimensional hydrogels were used to demonstrate the importance of mechanical memory on chondrogenic potential by comparing the threshold after exposure to a two-dimensional stiff substrate (Scott et al., 2023). Importantly, a recent study reported on the pivotal role of integrin-mediated adhesion in regulating the epigenetic landscape to restrict DC maturation (Guenther et al., 2021). Additionally, adhesion-mediated reprogramming led to less effective anti-tumor responses by DCs, further suggesting the critical role of mechanical cues in maintaining immune cell phenotype and function (Harjunpää et al., 2024).

Engineered scaffolds create the opportunity to control mechanosensing and exposure to biochemical signals via encapsulation to induce cellular processes such as neurogenic, chondrogenic, or osteogenic differentiation (Soltani et al., 2024; Peng, 2024; Zahedi Tehrani et al., 2024). In a study using chitosan hydrogel encapsulating chemotactic simvastatin and osteogenic pargyline, endogenous stem cells were successfully recruited to promote osteogenic differentiation and bone regeneration (Wan et al., 2022). This scaffold promoted in situ bone regeneration by inducing epigenetic changes akin to training in the MSCs.

**Figure 3.**
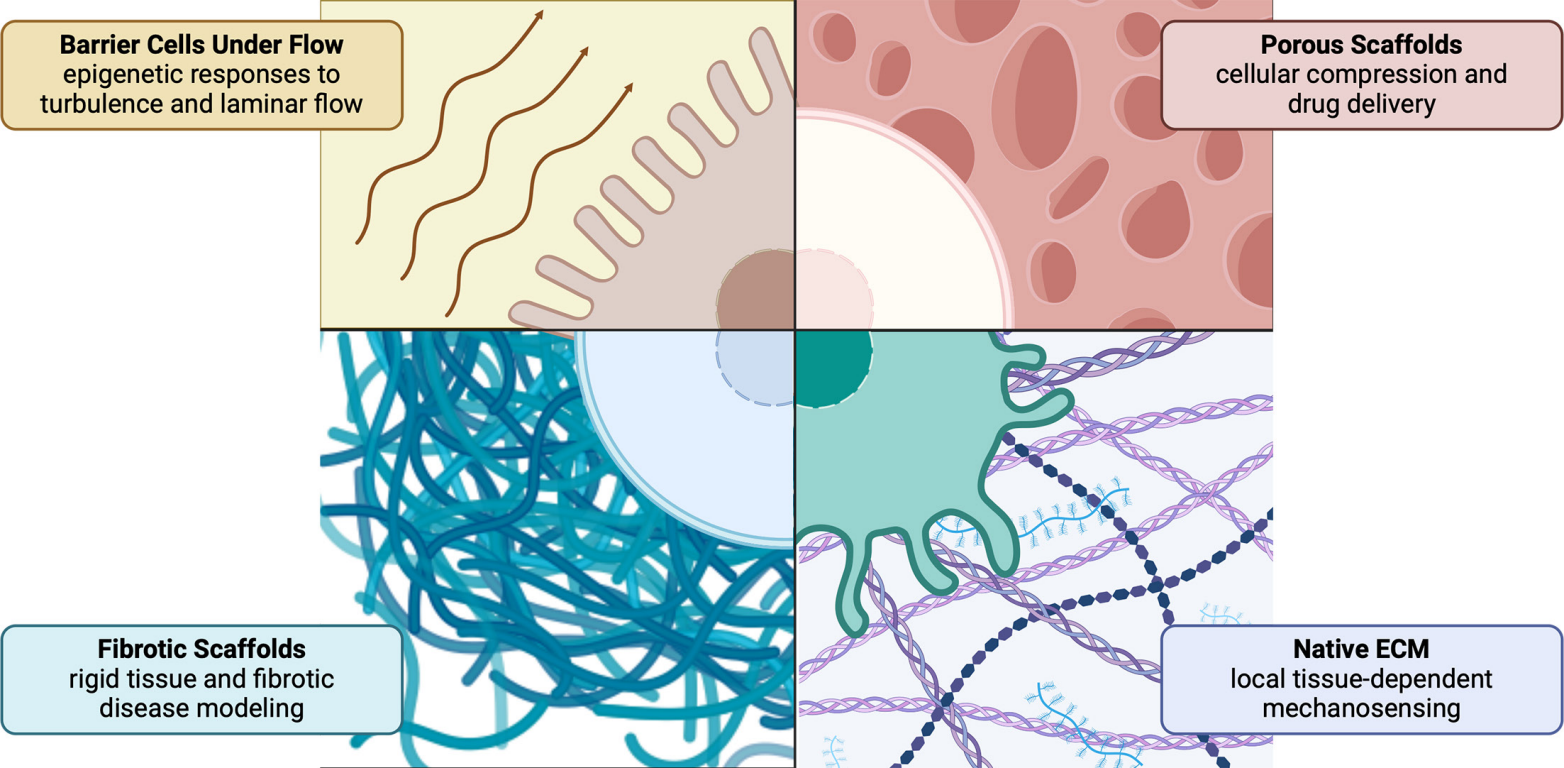
Types of biomechanical modulation achieved with native and engineered in vitro, ex vivo, and in vivo systems. Engineered scaffolds can model fluid flow, porous environments, fibrosis, and healthy extracellular matrices. Shear stress from turbulent fluid flow impacts endothelial cell susceptibility to atherosclerosis, likely due to trained immunity. Porous scaffolds can provide niches for cellular interaction, differentiation, and drug encapsulation. Fibrotic and native extracellular matrix (ECM), which exhibit differences in elasticity, stiffness, and ligand expression, can be used to measure the effects of mechanotransduction on training in healthy and diseased tissues. This figure was created with BioRender.com.

Bioengineered scaffolds can help answer many important questions related to trained immunity, including the epigenetic effects of relative stress, forces over a defined period of time, and the mechanics at interfaces and in three dimensions. Various platforms have been developed to study mechanotransduction, from simple coated cell culture plates to elaborate scaffolds (Figure 3). For example, collagen can be coated onto cell culture plates to study two-dimensional cell stretching, but also be used to fabricate three-dimensional gels to create in vivo-like matrices (Hackett et al., 2022). Specific tissues of interest can be modeled by using different polymers or manufacturing techniques. Crosslinking of chitosan and gelatin, followed by lyophilization yields highly porous scaffolds that can be seeded with MSCs to mimic bone tissue (Carvalho et al., 2024). Electrospinning of nanofibers can create ECM-like scaffolds and has high flexibility to incorporate other bioactive molecules. In a study using coaxial electrospinning, scaffolds were made of polycaprolactone and gelatin with zinc oxide and silicon dioxide nanoparticles to form a biomimetic periosteum. These scaffolds demonstrated high osteogenic differentiation ability and promoted an M2 phenotype of macrophages when seeded into the scaffolds (Zhuang et al., 2025).

Scaffolds can be engineered to have specific mechanical properties and can be put under different types of stress in vitro (Ni et al., 2023; Xu et al., 2022; Whitehead et al., 2018; Ramey-Ward et al., 2023). Specifically, cells can be put under shear stress from fluid flow. For cells like endothelial cells, shear stress-induced mechanotransduction is critical to some key molecules such as eNOS, an important target for prevention of atherosclerosis (Hecker et al., 1994). Cell culture can be set up under laminar flow, unidirectional flow to mimic healthy conditions, or disturbed flow to mimic hemodynamics prone to atherogenesis (Li et al., 2024).

Overall, these studies show scaffolds can induce epigenetic changes by providing mechanical cues and by acting as deposits of molecules that induce epigenetic reprogramming. Additionally, bioengineered scaffolds are inexpensive, easily modifiable tools to interrogate the role of mechanosensing in trained immunity. How these mechanical cues influence trained immunity in cells, both independently and in the context of chemical signals, remains a clear gap in knowledge that could be addressed with bioengineering approaches.

### Cellular engineering

Epigenetic and metabolic reprogramming are the principal mechanisms underlying the induction and maintenance of trained immunity. Epigenetic modification of histones, especially H3K4Me1, H3K4Me3, and H3K27Ac, increases the accessibility of chromatin in trained innate immune cells, thus improving their ability to rapidly transcribe effector proteins on demand (Novakovic et al., 2016; Saeed et al., 2014). This process is influenced by a set of long non-coding RNAs (lncRNAs) called immune gene priming lncRNAs (IPLs), which alter the 3D structure of the genome to facilitate epigenetic training (Fanucchi et al., 2019). Furthermore, transcription factors (TFs), including Jun and Fos, play a role in both the establishment and maintenance of training-induced epigenetic modifications (Larsen et al., 2021). Though it is a defining feature, epigenetic reprogramming is not the only hallmark of trained immunity. Alterations in cellular metabolism are necessary for the induction of training in a variety of model systems. Trained innate immune cells are more metabolically active than their untrained counterparts. The first known metabolic pathway demonstrated to be upregulated in trained immunity was glycolysis (Cheng et al., 2014). Blocking glycolysis using a non-hydrolyzable form of glucose inhibits training by BCG or β-glucan (Cheng et al., 2014; Arts et al., 2016a). Exploiting native cellular metabolism by modulating intermediate metabolites directly can also result in a trained phenotype in human monocytes, as in the case of mevalonate and itaconate (Bekkering et al., 2018; Domínguez-Andrés et al., 2019; Ferreira et al., 2023). This upregulation in glycolysis results in the accumulation of metabolic intermediates that are used as methyl- and acetyl-group sources for histone-modifying enzymes; therefore, the epigenetic and metabolic drivers of trained immunity are inherently linked (Arts et al., 2016b; Fanucchi et al., 2021; Riksen and Netea, 2021).

**Figure 4.**
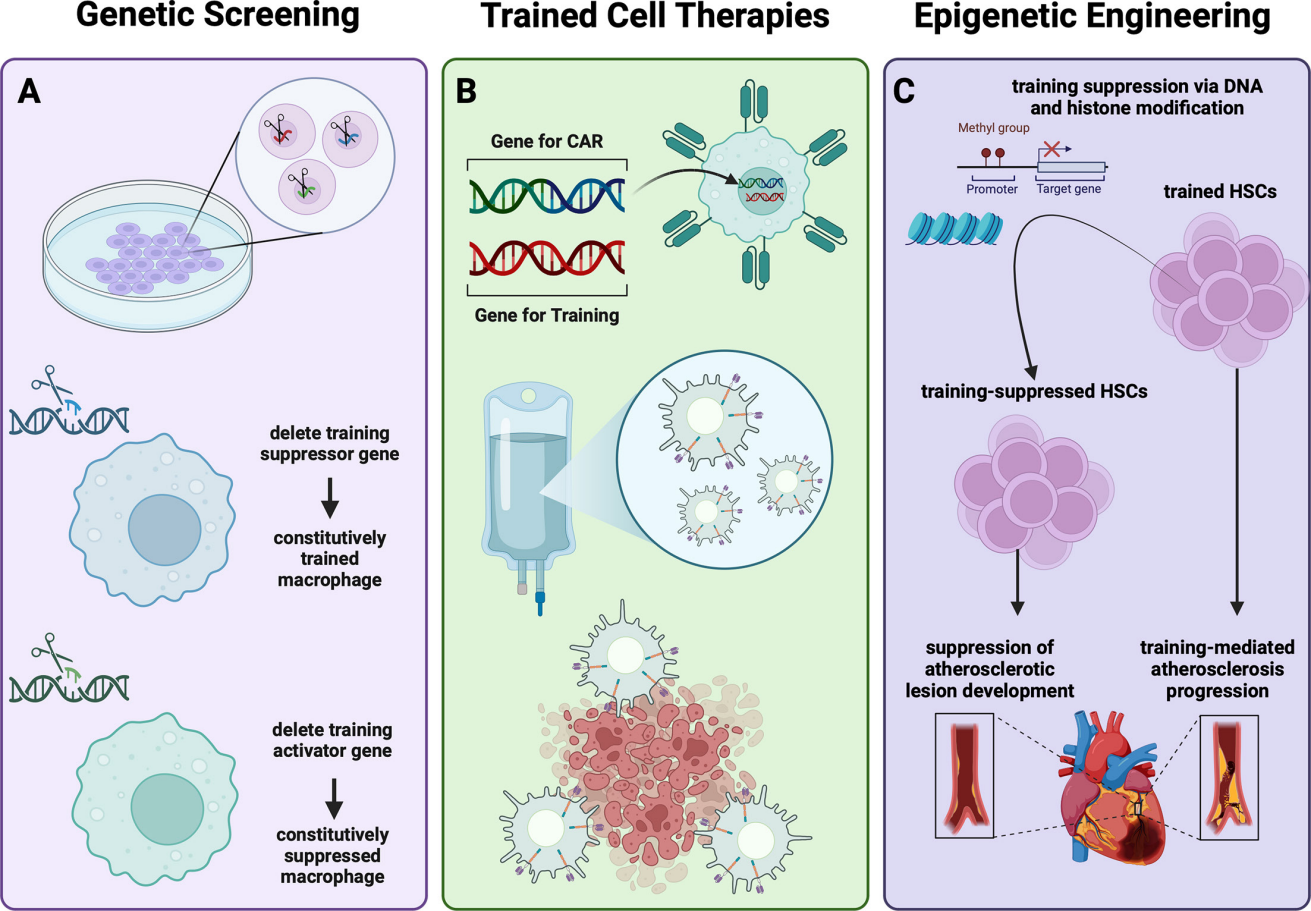
Methods and targets for cellular engineering of trained immunity. (**A**) As we use screening tools to elucidate the role of trained innate cells in additional autoimmune and inflammatory disorders, this concept will have increasingly more applications when designing therapeutics, including the activation and suppression of training programs. (**B**) Trained chimeric antigen receptor (CAR):-Macs generated ex vivo could resist immunosuppression of the tumor microenvironment to promote tumor cell death. (**C**) For example, trained immunity in atherosclerosis has been shown to contribute to disease pathogenesis and appears to be NLRP3 dependent (Netea et al., 2016; Moorlag et al., 2020). *Knocking down NLRP3 expression in patient macrophages could decrease disease burden*. This figure was created with BioRender.com.

The mechanisms underlying trained immunity are complex and intertwined, making it difficult to separate the contributions of individual pathways. Moreover, recent studies suggest changes in chromatin and RNA profiles in the context of trained immunity are stimulus-specific (Knight et al., 2024; O’Farrell et al., 2025). While these exciting discoveries suggest current therapies could be improved with disease-specific immune training programs, deeper mechanistic insight into stimulus-specific training pathways is needed. To deconstruct and interrogate the individual genetic pathways involved in trained immunity, high-throughput screening is an efficient and effective method. Two types of screens are frequently used to identify genetic programs contributing to trained phenotypes due to their efficiency and flexibility: small-molecule screens and single-guide RNA (sgRNA) CRISPR screens (Covarrubias et al., 2020). Small-molecule functional assays may be used to identify molecular pathways of interest and potential drugs simultaneously, but target identification remains an outstanding issue with this technique. Alternatively, sgRNA CRISPR knockout screening enables the identification of a specific target gene, but does not offer a potential therapeutic. Both small-molecule and CRISPR screens have identified genes involved in macrophage inflammatory pathways. As we begin to understand which genes, histone modifications, and metabolic pathways are required for training through screening techniques, deep sequencing, and metabolomics, novel clinical treatments for patients can be developed (Figure 4; John et al., 2022; Zhang et al., 2022).

Gene-editing technologies have improved drastically in the past decades, enabling targeted gene editing with few off-target effects in patients for the first time with CRISPR-Cas9 and lentiviral vectors (FDA News Release, 2023; Frangoul et al., 2024; FDA, 2022; Libmeldy, 2020). CRISPR-Cas9 systems use an RNA guide to position the Cas9 enzyme at the complementary gene on the DNA. The enzyme then cuts the DNA and the sequence of interest is either removed or replaced with a mutation or insertion, leading to a knockdown, or a gene replacement, respectively (Li et al., 2023b). A recent study by Tremblay et al., 2025 successfully used Cas9 epigenetic editing to reduce cholesterol levels in mice (Tremblay et al., 2025). Future applications may allow deposition or removal of specific training markers onto the DNA to modulate a training response. For example, it may be possible to suppress the glycolytic amplification of trained immunity by modulating the expression of key intermediate enzymes. By reducing the availability of metabolic intermediates, there could be a decrease in epigenetic modifications and therefore training in the affected cells.

A current challenge of this approach is the delivery process; CRISPR-Cas systems must be delivered to cells via viral vector, nanoparticles, electroporation, or another method prior to the introduction of sgRNA targeting the gene of interest. Alternatively, lentiviral vectors, or retroviruses engineered to prevent viral replication, can be used to deliver desired genetic materials to a cell directly, without the need for a Cas system. Lentiviral systems simplify delivery by encoding the gene of interest directly into the delivery agent. However, lentiviruses have fewer applications than CRISPR-Cas9, as control over their integration site is limited. Although specific retroviruses have preferences for certain insertion sites in the genome, variations in insertion location exist, increasing the likelihood of off-target effects and preventing directed gene knockdowns (Poletti and Mavilio, 2021).

Another approach to improve the delivery of genetic components to a specific cellular subset is autologous cell therapy (ACT). This approach involves removing the cell population of interest from a patient, manipulating the cells to have certain characteristics or gene-expression patterns, and infusing them back into the patient (El-Kadiry et al., 2021). By using gene-editing technologies in combination with ACT, key genes can be over- or under-expressed in harvested cells to return them to a state of healthy activity, ideally abating the disease phenotypes upon reinfusion. For instance, patients with immunodeficiencies may benefit from increased immune system activation via the overexpression of a core training gene in macrophages. Once a gene to induce the desired training phenotype is identified, this gene could be overexpressed in patient macrophages ex vivo with CRISPR-Cas9, and the trained cells could be reinfused into the patient, resulting in a decreased occurrence of infections.

Chimeric antigen receptor (CAR) macrophages are a newer ACT, analogous to CAR T cells. Immunocompromised patients may benefit from receiving such an ACT in which macrophages are produced with a trained phenotype before being infused back into the patient. CAR macrophages (also known as CAR-Macs) are currently in clinical trials for cancer treatment due to their ability to infiltrate the tumor microenvironment as tumor-associated macrophages (TAMs) (Reiss et al., 2025). Macrophages are processed ex vivo to express a tumor-specific receptor, becoming CAR-Macs that preferentially accumulate in the tumor. Training CAR-Macs before infusion could allow them to resist the immunosuppressive signals of the tumor microenvironment. By overexpressing key genes involved in training to increase immunogenicity, the tumor environment could be flipped from ‘cold’ and immunosuppressive to ‘hot’ and immunocompetent, thus improving patient outcomes. In summary, cellular engineering can deepen our fundamental understanding of trained immunity by identifying the core genes underlying the training mechanism and expand therapeutic applications of innate training by direct cellular manipulation.

### Computational systems and machine learning

During the past several years, machine learning tools and their applications have revolutionized drug discovery and mechanistic research. Trained immunity, with its history rooted in large, complex datasets, is uniquely poised to benefit from these approaches. Particularly, bioinformatics and machine learning systems can be used to identify evidence of training in epidemiologic and clinical datasets, integrate training signals across omics platforms, and identify novel pathways and targets for investigation.

**Figure 5.**
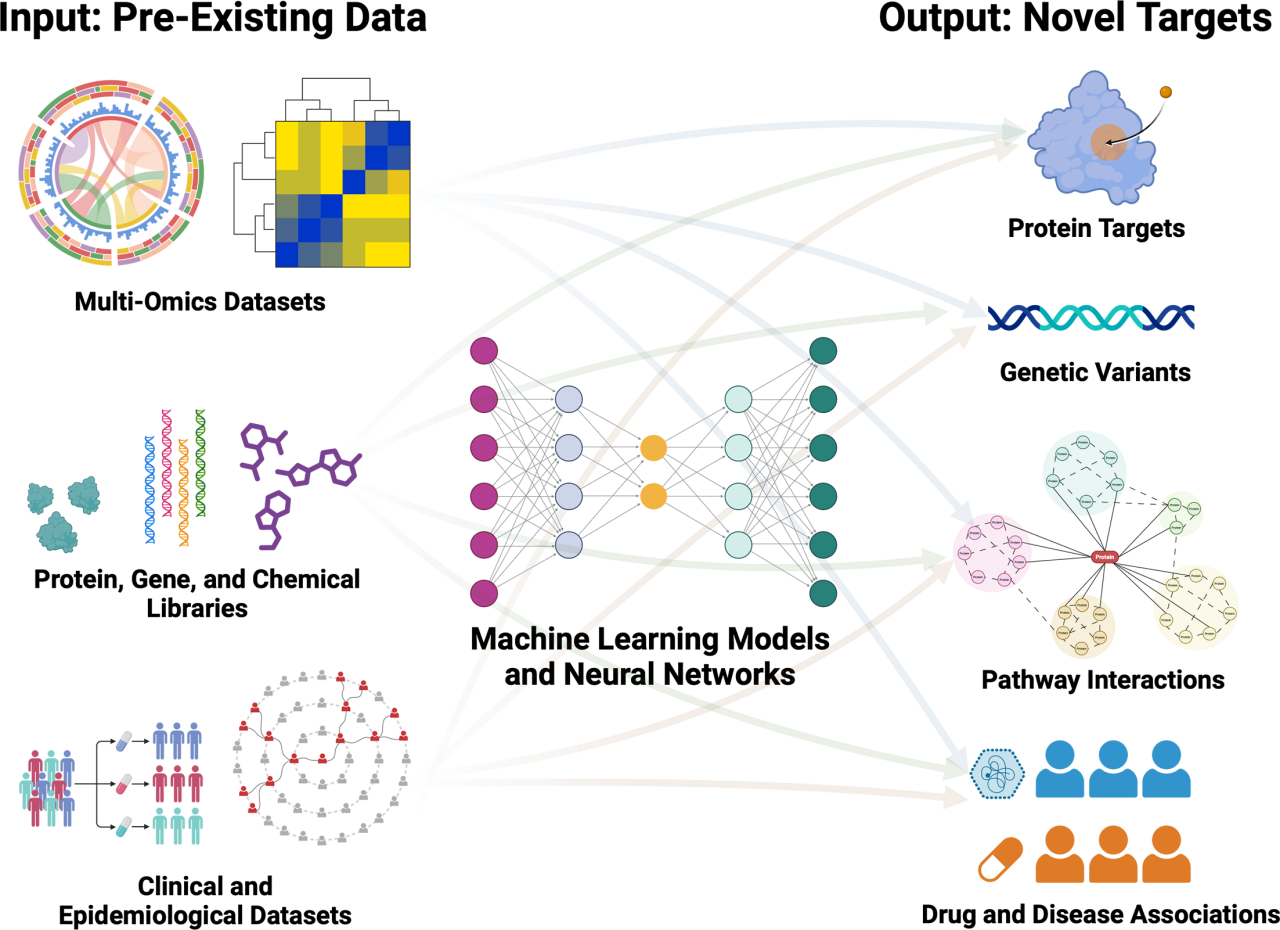
Pre-existing data sources for machine learning-based discovery in trained immunity. Sequencing datasets, including transcriptomics, epigenomics, and translatomics can be integrated to determine the effects of intracellular regulation on trained immunity effector responses. A comparison of chemical and protein libraries with known training pathways can identify protein targets, pathways, and potential mechanisms for novel induction of trained immunity. Epidemiological and clinical datasets could yield particularly rich information, including the influence of genetic variants, microbiota, drugs, and disease states on trained immunity. This figure was created with BioRender.com.

Trained immunity was first described after infants vaccinated with Bacille Calmette-Guerin (BCG) displayed non-specific disease resistance, resulting in reduced infant mortality (Aaby et al., 2011). The identification of additional trained immunity inducers from the existing pool of vaccines is limited by current vaccine approval guidelines, which do not require routine assessment of non-specific vaccine effects. Benn et al., 2023 proposed a novel regulatory framework for vaccine approval and regulation, which requires a holistic review of overall mortality and morbidity for all infectious diseases throughout a phase III trial (Benn et al., 2023). Reliable reporting of non-specific vaccine effects (e.g. unrelated infections, inflammatory conditions, and all-cause mortality) would enable the establishment of a large epidemiological data pool with which a data-driven machine learning (ML) model can be trained to detect statistical patterns related to infection and inflammatory disease risk independent of vaccine-specific immunity. The development of such a deep learning architecture, such as this would produce an additional avenue to identify trained immunity inducers, like BCG, and further characterize training-related heterologous effects in patient populations (Figure 5). An analogous deep-learning model trained to extract complex patterns and correlations from high-dimensional data encompassing trained immunity-related changes in chromatin accessibility, cellular metabolism, and inflammatory cytokine production from existing immunological data sets could also pinpoint current vaccines, biologics, and other pharmaceutical agents that are likely to induce innate immune training (Tang et al., 2023; Bravi, 2024; Ochando et al., 2023).

Utilizing machine learning to identify or predict the occurrence of trained immunity has become increasingly feasible due to ongoing efforts to elucidate the underlying molecular and cellular mechanisms of trained immunity. Training-induced alterations in the accessibility of chromatin regions linked to glycolysis and mRNA processing are well characterized (Cheng et al., 2014; Arts et al., 2016a). Bock and colleagues (2024) exploited chromatin accessibility patterns present in their multi-omics data to train a ML model capable of predicting whether an individual would respond to BCG as a training stimulus based on their baseline chromatin accessibility profile (Moorlag et al., 2024). Therefore, accessing the individual efficacy of any future trained immunity-based therapeutics is feasible. This advancement could be achieved by developing a deep learning ML model that is trained to predict which stimulus-specific physiological changes would display the highest efficacy in a specific population (O’Farrell et al., 2025). In addition to predicting therapeutic efficacy, ML-guided genomics expression and variant analysis can be used to identify novel protein targets via pathway enrichment analysis. Incorporating known protein targets and their associated genomic regulation can also aid in elucidating novel molecular connections within relevant biological networks (Mallick et al., 2023). Predicted protein targets can be further validated by evaluating their involvement in known training-associated physiological processes such as chromatin organization, long non-coding RNA transcription, DNA methylation, and cellular metabolism (Netea et al., 2020).

The introduction of training-based therapies into clinical practice can be accelerated by employing in silico techniques in tandem with ML models to drive therapeutic discovery. Understanding the intricacies of protein structure, protein-protein interactions, and protein-ligand interactions has been made possible with ML tools such as AlphaFold3 and RoseTTA Fold (Baek et al., 2021; Abramson et al., 2024). The advancement of such in silico methods expedites drug discovery by providing increasingly accurate predictions which minimize the experimental effort required to identify and develop drug candidates. For example, these advancements allowed for the de novo protein design of small protein binders to an important innate immune cell activator, TLR3 (Adams et al., 2025). Deep learning-based large language models such as PrefixProt and ProGen could be utilized to design a specialized protein-based therapy, such as a constitutively active metabolic enzyme that produces metabolic intermediates, like mevalonate or itaconate, which are known to induce training (Madani et al., 2023; Guan et al., 2025; Luo et al., 2024).

The determination of druggable protein targets for trained immunity induction can be expedited by utilizing known small-molecule inducers for mechanistic interrogation (Knight et al., 2024; Ajit et al., 2024). Unlike endogenous signalling molecules and pathogen-derived sources, such as BCG and β-glucan, which have been used for mechanistic interrogation thus far, the use of small molecules is well precedented in in silico ligand-based target prediction tools such as SwissTargetPrediction and TargetHunter (Wang et al., 2013; Yang et al., 2023; Li et al., 2017; Daina et al., 2019). In addition to providing additional in silico and experimental methods for druggable target prediction, small molecule-based mechanistic interrogation creates an avenue for which peripheral or central trained immunity-specific targets can be recognized by an artificial neural network (ANN)-based machine learning architecture (Liao et al., 2022). Additional small molecule training inducers could be identified with data-driven ML models, which systematically guide high-throughput molecular screening via quantitative structure-activity relationship (QSAR) study (Bernatavicius et al., 2024; Niazi and Mariam, 2023). With limited small-molecule screening data, a deep representational learning-based QSAR framework can identify candidate molecules by iterative feedback from additional experimental data and molecular docking simulations (Tang et al., 2023). While it is still unclear where trained immunity and machine learning will intersect, the many opportunities created by these computational models offer exciting new areas of research in immunology and engineering.

### Conclusions and future directions

As our understanding of the applications of trained immunity continues to grow, new approaches are needed to fully explore the therapeutic and fundamental aspects of training, which are inextricably connected. While the implications of trained immunity in the context of autoinflammatory and autoimmune disease are rapidly expanding, practical approaches to suppress maladaptive training and engage the beneficial applications of training in human diseases are still lacking.

#### Looking towards the future: the clinical state of trained immunity therapeutics

While there are clear clinical implications for applying trained immunity to the treatment of disease, most new human studies on trained immunity are early-stage observational studies with descriptive in vitro endpoints rather than interventional clinical trials. For example, current clinical studies on trained immunity in the recruitment phase include: vaccination with live vaccines including BCG, MMR (Eklöf, 2024), and yellow fever (Institut Pasteur, 2024), and inactivated tuberculosis vaccine MV130 Inmunotek, 2024; impact of sleep disorders on trained immunity in the context of COVID-19 Freixinet, 2024; impact of pheochromocytoma (Radboud University Medical Center, 2024), colon carcinoma, and thyroid carcinoma (Radboud University Medical Center, 2023) on trained immunity with or without ex vivo exposure to additional training stimuli; and effect of statin treatment on training state in patients with high cholesterol (Cheng, 2023). These studies often interrogate differences in the immune response of peripheral mononuclear cells of different patient populations, with or without certain conditions or treatments, to determine which population exhibits enhanced innate responses, without evaluating functional changes in disease processes. While these studies provide useful data and are minimally invasive to the study participants, this strategy is a major limitation of current clinical applications of trained immunity, as it is difficult to make strong claims about clinical efficacy of trained immunity from observational studies alone. Additionally, methods to suppress or reverse maladaptive central trained immunity in chronic inflammatory disease are currently understudied in clinical settings, which is unsurprising as the link between training and comorbid autoinflammatory conditions was only made in the last decade. Furthermore, with the new understanding that training responses are stimulus-specific, stimuli beyond BCG and β-glucan will be required to access other forms of trained immunity for specific applications. Future studies involving direct intervention for the modulation and prevention of disease are needed to provide clinical evidence for training-based therapies.

#### Bioengineered therapies and the potential for combinatorial training regimens

Bioengineering-based therapies, however, are a major focus of ongoing interventional clinical trials across multiple disease areas, including cancer, tissue grafting, and chronic disease. Currently, recruiting trials using bioengineering approaches include: tissue engineering for arthritis (University Hospital, Basel, Switzerland, 2025), skin grafts (Ma, 2025; Jiayuan, 2020; Andalusian Network for Design and Translation of Advanced Therapies, 2023), and breast reconstruction Quanta Medical, 2025; engineered T-cells for chronic kidney disease (Centre Hospitalier Régional d’Orléans, 2024) and advanced solid tumors Therapeutics, 2024; and allograft for periodontal regeneration (Pulido, 2024), among many others. Each of these cases represents unique opportunities for integrating trained immunity into cutting-edge therapeutic applications. Autologous or allograft-engineered tissue may benefit from the training of epithelial cells and progenitors to enhance tissue healing and prevent graft rejection. Trained, engineered macrophages could be co-administered with engineered T-cells to enhance engraftment and efficacy in cancer or chronic kidney disease. Finally, periodontal disease has known effects on trained immunity, so integrating training suppressors alongside an allograft could mitigate the risk of concurrent autoinflammatory disease. In these systems, trained immunity may serve as adjuvant therapy to enhance the efficacy of bioengineered therapeutics.

By implementing trained immunity alongside well-tested bioengineering interventions in combinatorial approaches, trained immunity is more likely to exhibit clinically significant success. For many diseases of interest, especially cancer, engaging the innate immune system alone via trained immunity may be limited in its potential to control disease, making a combinatorial approach that also activates adaptive immunity or limits disease pathogenesis more preferable. The combinatorial approach is already in use for one training-based therapy: intravesicular BCG is commonly used as adjuvant therapy following surgical resection for the treatment of bladder cancer (Alexandroff et al., 1999; Buffen et al., 2014). Additionally, combinatorial therapeutic approaches are often the preferred clinical approach when evaluating experimental treatments in cancer, to mitigate both ethical and efficacy concerns.

#### Conclusions

In this review, we propose that the strategic application of bioengineering-derived techniques would allow us to refine the activation of trained immunity for safer, more localized, and more effective training-based therapeutics. For localization, nanotherapeutics are modular, well-studied systems that can precisely deliver inducers of trained immunity to select tissues. For effectiveness, understanding the biomechanics of training at a cellular level will influence our approach to studying and manipulating trained cells in both in vivo and ex vivo experimental systems and therapeutics. For clinical translation, using training in cell-based therapies is a key next step to ensure these highly effective, costly therapeutics retain their function in suppressive disease microenvironments. Conversely, using cellular engineering to turn off training in hyperinflammatory states could slow the progression of diseases like atherosclerosis. Looking toward the near future, machine learning can identify trained immunity phenotypes in pre-existing, multi-omic, and clinical datasets. Employing ML models to determine where training already occurs will improve our understanding of the landscape of training, both in detrimental and protective cases. Bioengineering will be both necessary and sufficient to transition trained immunity from scientific observation to interventional therapeutics.

## References

[bib1] Aaby P, Roth A, Ravn H, Napirna BM, Rodrigues A, Lisse IM, Stensballe L, Diness BR, Lausch KR, Lund N, Biering-Sørensen S, Whittle H, Benn CS (2011). Randomized trial of BCG vaccination at birth to low-birth-weight children: beneficial nonspecific effects in the neonatal period?. The Journal of Infectious Diseases.

[bib2] Abramson J, Adler J, Dunger J, Evans R, Green T, Pritzel A, Ronneberger O, Willmore L, Ballard AJ, Bambrick J, Bodenstein SW, Evans DA, Hung C-C, O’Neill M, Reiman D, Tunyasuvunakool K, Wu Z, Žemgulytė A, Arvaniti E, Beattie C, Bertolli O, Bridgland A, Cherepanov A, Congreve M, Cowen-Rivers AI, Cowie A, Figurnov M, Fuchs FB, Gladman H, Jain R, Khan YA, Low CMR, Perlin K, Potapenko A, Savy P, Singh S, Stecula A, Thillaisundaram A, Tong C, Yakneen S, Zhong ED, Zielinski M, Žídek A, Bapst V, Kohli P, Jaderberg M, Hassabis D, Jumper JM (2024). Accurate structure prediction of biomolecular interactions with AlphaFold 3. Nature.

[bib3] Adams CS, Kim H, Burtner AE, Lee DS, Dobbins C, Criswell C, Coventry B, Tran-Pearson A, Kim HM, King NP (2025). De novo design of protein minibinder agonists of TLR3. Nature Communications.

[bib4] Ajit J, Cassaidy B, Tang S, Solanki A, Chen Q, Shen J, Esser Kahn AP (2022). Temporal control of trained immunity via encapsulated release of β-Glucan improves therapeutic applications. Advanced Healthcare Materials.

[bib5] Ajit J, Knight HR, Chen Q, Solanki A, Shen J, Kahn APE (2024). Novel Non-Immunogenic Trained Immunity Inducing Small Molecule with Improved Anti-Tumor Propertie. bioRxiv.

[bib6] Alexandroff AB, Jackson AM, O’Donnell MA, James K (1999). BCG immunotherapy of bladder cancer: 20 years on. Lancet.

[bib7] Andalusian Network for Design and Translation of Advanced Therapies (2023). Phase II Clinical Trial Evaluating the Safety and Efficacy of a Tissue Engineered Autologous Skin Substitute Reconstructive Surgery for Basal Cell Carcinoma. Clinicaltrials.gov.

[bib8] Aragona M, Panciera T, Manfrin A (2013). A mechanical checkpoint controls multicellular growth through YAP/TAZ regulation by actin-processing factors. Cell.

[bib9] Arias JL, Unciti-Broceta JD, Maceira J, del Castillo T, Hernández-Quero J, Magez S, Soriano M, García-Salcedo JA (2015). Nanobody conjugated PLGA nanoparticles for active targeting of African Trypanosomiasis. Journal of Controlled Release.

[bib10] Arts RJW, Carvalho A, La Rocca C, Palma C, Rodrigues F, Silvestre R, Kleinnijenhuis J, Lachmandas E, Gonçalves LG, Belinha A, Cunha C, Oosting M, Joosten LAB, Matarese G, van Crevel R, Netea MG (2016a). Immunometabolic pathways in BCG-induced trained immunity. Cell Reports.

[bib11] Arts RJW, Novakovic B, Ter Horst R, Carvalho A, Bekkering S, Lachmandas E, Rodrigues F, Silvestre R, Cheng S-C, Wang S-Y, Habibi E, Gonçalves LG, Mesquita I, Cunha C, van Laarhoven A, van de Veerdonk FL, Williams DL, van der Meer JWM, Logie C, O’Neill LA, Dinarello CA, Riksen NP, van Crevel R, Clish C, Notebaart RA, Joosten LAB, Stunnenberg HG, Xavier RJ, Netea MG (2016b). Glutaminolysis and fumarate accumulation integrate immunometabolic and epigenetic programs in trained immunity. Cell Metabolism.

[bib12] Arts RJW, Moorlag SJCFM, Novakovic B, Li Y, Wang S-Y, Oosting M, Kumar V, Xavier RJ, Wijmenga C, Joosten LAB, Reusken CBEM, Benn CS, Aaby P, Koopmans MP, Stunnenberg HG, van Crevel R, Netea MG (2018). BCG Vaccination protects against experimental viral infection in humans through the induction of cytokines associated with trained immunity. Cell Host & Microbe.

[bib13] Augustine R, Hasan A, Primavera R, Wilson RJ, Thakor AS, Kevadiya BD (2020). Cellular uptake and retention of nanoparticles: Insights on particle properties and interaction with cellular components. Materials Today Communications.

[bib14] Baek M, DiMaio F, Anishchenko I, Dauparas J, Ovchinnikov S, Lee GR, Wang J, Cong Q, Kinch LN, Schaeffer RD, Millán C, Park H, Adams C, Glassman CR, DeGiovanni A, Pereira JH, Rodrigues AV, van Dijk AA, Ebrecht AC, Opperman DJ, Sagmeister T, Buhlheller C, Pavkov-Keller T, Rathinaswamy MK, Dalwadi U, Yip CK, Burke JE, Garcia KC, Grishin NV, Adams PD, Read RJ, Baker D (2021). Accurate prediction of protein structures and interactions using a three-track neural network. Science.

[bib15] Bajracharya R, Song JG, Patil BR, Lee SH, Noh H-M, Kim D-H, Kim G-L, Seo S-H, Park J-W, Jeong SH, Lee CH, Han H-K (2022). Functional ligands for improving anticancer drug therapy: current status and applications to drug delivery systems. Drug Delivery.

[bib16] Barenholz Y (2012). Doxil — The first FDA-approved nano-drug: lessons learned. Journal of Controlled Release.

[bib17] Bautista-Hernández LA, Gómez-Olivares JL, Buentello-Volante B, Bautista-de Lucio VM (2017). Fibroblasts: the unknown sentinels eliciting immune responses against microorganisms. European Journal of Microbiology and Immunology.

[bib18] Bekkering S, van den Munckhof I, Nielen T, Lamfers E, Dinarello C, Rutten J, de Graaf J, Joosten LAB, Netea MG, Gomes MER, Riksen NP (2016). Innate immune cell activation and epigenetic remodeling in symptomatic and asymptomatic atherosclerosis in humans in vivo. Atherosclerosis.

[bib19] Bekkering S, Arts RJW, Novakovic B, Kourtzelis I, van der Heijden C, Li Y, Popa CD, Ter Horst R, van Tuijl J, Netea-Maier RT, van de Veerdonk FL, Chavakis T, Joosten LAB, van der Meer JWM, Stunnenberg H, Riksen NP, Netea MG (2018). Metabolic induction of trained immunity through the mevalonate pathway. Cell.

[bib20] Benn CS, Netea MG, Selin LK, Aaby P (2013). A small jab - a big effect: nonspecific immunomodulation by vaccines. Trends in Immunology.

[bib21] Benn CS, Amenyogbe N, Björkman A, Domínguez-Andrés J, Fish EN, Flanagan KL, Klein SL, Kollmann TR, Kyvik KO, Netea MG, Rod NH, Schaltz-Buchholzer F, Shann F, Selin L, Thysen SM, Aaby P (2023). Implications of non-specific effects for testing, approving, and regulating vaccines. Drug Safety.

[bib22] Bernatavicius A, Šícho M, Janssen APA, Hassen AK, Preuss M, van Westen GJP (2024). Alphafold meets de novo drug design: leveraging structural protein information in multitarget molecular generative models. Journal of Chemical Information and Modeling.

[bib23] Blossey AM, Brückner S, May M, Parzmair GP, Sharma H, Shaligram U, Grode L, Kaufmann SHE, Netea MG, Schindler C (2023). VPM1002 as prophylaxis against severe respiratory tract infections including coronavirus disease 2019 in the elderly: a phase 3 randomized, double-blind, placebo-controlled, multicenter clinical study. Clinical Infectious Diseases.

[bib24] Bravi B (2024). Development and use of machine learning algorithms in vaccine target selection. NPJ Vaccines.

[bib25] Buffen K, Oosting M, Quintin J, Ng A, Kleinnijenhuis J, Kumar V, van de Vosse E, Wijmenga C, van Crevel R, Oosterwijk E, Grotenhuis AJ, Vermeulen SH, Kiemeney LA, van de Veerdonk FL, Chamilos G, Xavier RJ, van der Meer JWM, Netea MG, Joosten LAB (2014). Autophagy controls BCG-induced trained immunity and the response to intravesical BCG therapy for bladder cancer. PLOS Pathogens.

[bib26] Cafri G, Gartner JJ, Zaks T, Hopson K, Levin N, Paria BC, Parkhurst MR, Yossef R, Lowery FJ, Jafferji MS, Prickett TD, Goff SL, McGowan CT, Seitter S, Shindorf ML, Parikh A, Chatani PD, Robbins PF, Rosenberg SA (2020). mRNA vaccine-induced neoantigen-specific T cell immunity in patients with gastrointestinal cancer. The Journal of Clinical Investigation.

[bib27] Carvalho JA, Jayme CC, Matsuo FS, Tedesco AC (2024). Chitosan‐gelatin/hydroxyapatite‐based scaffold associated with mesenchymal stem cells differentiate into osteoblasts improves the surface of the bone lesion in mice C57BL/6J. Journal of Applied Polymer Science.

[bib28] Centre Hospitalier Régional d’Orléans (2024). Assessment of T-Cell Response and In-Vitro Proof-of-Concept of T-Cell Engineering in Chronic End-Stage Kidney Disease Patients. Clinicaltrials.gov.

[bib29] Champion JA, Mitragotri S (2006). Role of target geometry in phagocytosis. PNAS.

[bib30] Chavakis T, Mitroulis I, Hajishengallis G (2019). Hematopoietic progenitor cells as integrative hubs for adaptation to and fine-tuning of inflammation. Nature Immunology.

[bib31] Cheng S-C, Quintin J, Cramer RA, Shepardson KM, Saeed S, Kumar V, Giamarellos-Bourboulis EJ, Martens JHA, Rao NA, Aghajanirefah A, Manjeri GR, Li Y, Ifrim DC, Arts RJW, van der Veer BMJW, Deen PMT, Logie C, O’Neill LA, Willems P, van de Veerdonk FL, van der Meer JWM, Ng A, Joosten LAB, Wijmenga C, Stunnenberg HG, Xavier RJ, Netea MG (2014). mTOR- and HIF-1α-mediated aerobic glycolysis as metabolic basis for trained immunity. Science.

[bib32] Cheng X (2023). Continuous Atorvastatin Therapy Compared With Intermittent Atorvastatin Therapy for the Effect of LDL-c Level Variability and the Regulation of Trained Immunity. Clinicaltrials.gov.

[bib33] Choudhury RP, Rong JX, Trogan E, Elmalem VI, Dansky HM, Breslow JL, Witztum JL, Fallon JT, Fisher EA (2004). High-density lipoproteins retard the progression of atherosclerosis and favorably remodel lesions without suppressing indices of inflammation or oxidation. Arteriosclerosis, Thrombosis, and Vascular Biology.

[bib34] Choudhury RP, Edgar L, Rydén M, Fisher EA (2021). Diabetes and metabolic drivers of trained immunity: new therapeutic targets beyond glucose. Arteriosclerosis, Thrombosis, and Vascular Biology.

[bib35] Christ A, Günther P, Lauterbach MAR, Duewell P, Biswas D, Pelka K, Scholz CJ, Oosting M, Haendler K, Baßler K, Klee K, Schulte-Schrepping J, Ulas T, Moorlag SJCFM, Kumar V, Park MH, Joosten LAB, Groh LA, Riksen NP, Espevik T, Schlitzer A, Li Y, Fitzgerald ML, Netea MG, Schultze JL, Latz E (2018). Western diet triggers NLRP3-dependent innate immune reprogramming. Cell.

[bib36] Citron P, Nerem RM (2004). Bioengineering: 25 years of progress—but still only a beginning. Technology in Society.

[bib37] Colombo M, Raposo G, Théry C (2014). Biogenesis, secretion, and intercellular interactions of exosomes and other extracellular vesicles. Annual Review of Cell and Developmental Biology.

[bib38] Covarrubias S, Vollmers AC, Capili A, Boettcher M, Shulkin A, Correa MR, Halasz H, Robinson EK, O’Briain L, Vollmers C, Blau J, Katzman S, McManus MT, Carpenter S (2020). High-Throughput CRISPR screening identifies genes involved in macrophage viability and inflammatory pathways. Cell Reports.

[bib39] Cox DBT, Platt RJ, Zhang F (2015). Therapeutic genome editing: prospects and challenges. Nature Medicine.

[bib40] Daina A, Michielin O, Zoete V (2019). SwissTargetPrediction: updated data and new features for efficient prediction of protein targets of small molecules. Nucleic Acids Research.

[bib41] Damiano MG, Mutharasan RK, Tripathy S, McMahon KM, Thaxton CS (2013). Templated high density lipoprotein nanoparticles as potential therapies and for molecular delivery. Advanced Drug Delivery Reviews.

[bib42] de Laval B, Maurizio J, Kandalla PK, Brisou G, Simonnet L, Huber C, Gimenez G, Matcovitch-Natan O, Reinhardt S, David E, Mildner A, Leutz A, Nadel B, Bordi C, Amit I, Sarrazin S, Sieweke MH (2020). C/EBPβ-dependent epigenetic memory induces trained immunity in hematopoietic stem cells. Cell Stem Cell.

[bib43] Del Fresno C, García-Arriaza J, Martínez-Cano S (2021). The bacterial mucosal immunotherapy MV130 protects against SARS-CoV-2 infection and improves COVID-19 vaccines immunogenicity. Frontiers in Immunology.

[bib44] Ding C, Shrestha R, Zhu X, Geller AE, Wu S, Woeste MR, Li W, Wang H, Yuan F, Xu R, Chariker JH, Hu X, Li H, Tieri D, Zhang H-G, Rouchka EC, Mitchell R, Siskind LJ, Zhang X, Xu XG, McMasters KM, Yu Y, Yan J (2023). Inducing trained immunity in pro-metastatic macrophages to control tumor metastasis. Nature Immunology.

[bib45] Domínguez-Andrés J, Novakovic B, Li Y, Scicluna BP, Gresnigt MS, Arts RJW, Oosting M, Moorlag SJCFM, Groh LA, Zwaag J, Koch RM, Ter Horst R, Joosten LAB, Wijmenga C, Michelucci A, van der Poll T, Kox M, Pickkers P, Kumar V, Stunnenberg H, Netea MG (2019). The itaconate pathway is a central regulatory node linking innate immune tolerance and trained immunity. Cell Metabolism.

[bib46] Dong Z, Hou L, Luo W, Pan L-H, Li X, Tan H-P, Wu R-D, Lu H, Yao K, Mu M-D, Gao C-S, Weng X-Y, Ge J-B (2024). Myocardial infarction drives trained immunity of monocytes, accelerating atherosclerosis. European Heart Journal.

[bib47] Duan Z, Luo Y (2021). Targeting macrophages in cancer immunotherapy. Signal Transduction and Targeted Therapy.

[bib48] Edgar L, Akbar N, Braithwaite AT, Krausgruber T, Gallart-Ayala H, Bailey J, Corbin AL, Khoyratty TE, Chai JT, Alkhalil M, Rendeiro AF, Ziberna K, Arya R, Cahill TJ, Bock C, Laurencikiene J, Crabtree MJ, Lemieux ME, Riksen NP, Netea MG, Wheelock CE, Channon KM, Rydén M, Udalova IA, Carnicer R, Choudhury RP (2021). Hyperglycemia induces trained immunity in macrophages and their precursors and promotes atherosclerosis. Circulation.

[bib49] Eklöf J (2024). Using Live Vaccines to Induce Beneficial Innate Immune Training and Reduce Systemic Inflammation in COPD Patients. Clinicaltrials.gov.

[bib50] El-Kadiry AEH, Rafei M, Shammaa R (2021). Cell therapy: types, regulation, and clinical benefits. Frontiers in Medicine.

[bib51] Ellipilli S, Wang H, Binzel DW, Shu D, Guo P (2023). Ligand-displaying-exosomes using RNA nanotechnology for targeted delivery of multi-specific drugs for liver cancer regression. Nanomedicine.

[bib52] Elosegui-Artola A, Andreu I, Beedle AEM, Lezamiz A, Uroz M, Kosmalska AJ, Oria R, Kechagia JZ, Rico-Lastres P, Le Roux A-L, Shanahan CM, Trepat X, Navajas D, Garcia-Manyes S, Roca-Cusachs P (2017). Force triggers YAP nuclear entry by regulating transport across nuclear pores. Cell.

[bib53] Engler AJ, Sen S, Sweeney HL, Discher DE (2006). Matrix elasticity directs stem cell lineage specification. Cell.

[bib54] Fanucchi S, Fok ET, Dalla E, Shibayama Y, Börner K, Chang EY, Stoychev S, Imakaev M, Grimm D, Wang KC, Li G, Sung WK, Mhlanga MM (2019). Immune genes are primed for robust transcription by proximal long noncoding RNAs located in nuclear compartments. Nature Genetics.

[bib55] Fanucchi S, Domínguez-Andrés J, Joosten LAB, Netea MG, Mhlanga MM (2021). The intersection of epigenetics and metabolism in trained immunity. Immunity.

[bib56] FDA (2022). ZYNTEGLO. https://www.fda.gov/vaccines-blood-biologics/zynteglo.

[bib57] FDA News Release (2023). FDA Approves First Gene Therapies to Treat Patients with Sickle Cell Disease. https://www.fda.gov/news-events/press-announcements/fda-approves-first-gene-therapies-treat-patients-sickle-cell-disease.

[bib58] Feng Y, Guo M, Zhao H, Han S, Dong Q, Cui M (2020). Mesenchymal-stem-cell-derived extracellular vesicles mitigate trained immunity in the brain. Frontiers in Bioengineering and Biotechnology.

[bib59] Ferreira AV, Kostidis S, Groh LA, Koeken VACM, Bruno M, Baydemir I, Kilic G, Bulut Ö, Andriopoulou T, Spanou V, Synodinou KD, Gkavogianni T, Moorlag SJCFM, Charlotte de Bree L, Mourits VP, Matzaraki V, Koopman WJH, van de Veerdonk FL, Renieris G, Giera M, Giamarellos-Bourboulis EJ, Novakovic B, Domínguez-Andrés J (2023). Dimethyl itaconate induces long-term innate immune responses and confers protection against infection. Cell Reports.

[bib60] Flavell SJ, Hou TZ, Lax S, Filer AD, Salmon M, Buckley CD (2008). Fibroblasts as novel therapeutic targets in chronic inflammation. British Journal of Pharmacology.

[bib61] Frangoul H, Locatelli F, Sharma A, Bhatia M, Mapara M, Molinari L, Wall D, Liem RI, Telfer P, Shah AJ, Cavazzana M, Corbacioglu S, Rondelli D, Meisel R, Dedeken L, Lobitz S, de Montalembert M, Steinberg MH, Walters MC, Eckrich MJ, Imren S, Bower L, Simard C, Zhou W, Xuan F, Morrow PK, Hobbs WE, Grupp SA, CLIMB SCD-121 Study Group (2024). Exagamglogene autotemcel for severe sickle cell disease. The New England Journal of Medicine.

[bib62] Freixinet AG (2024). Impact of Sleep Disorders on Innate Immunity in COVID-19 Patients. A Cohort Study. Clinicaltrials.gov.

[bib63] Geiger B, Spatz JP, Bershadsky AD (2009). Environmental sensing through focal adhesions. Nature Reviews. Molecular Cell Biology.

[bib64] Geller AE, Shrestha R, Woeste MR, Guo H, Hu X, Ding C, Andreeva K, Chariker JH, Zhou M, Tieri D, Watson CT, Mitchell RA, Zhang H-G, Li Y, Martin Ii RCG, Rouchka EC, Yan J (2022). The induction of peripheral trained immunity in the pancreas incites anti-tumor activity to control pancreatic cancer progression. Nature Communications.

[bib65] Giamarellos-Bourboulis EJ, Tsilika M, Moorlag S, Antonakos N, Kotsaki A, Domínguez-Andrés J, Kyriazopoulou E, Gkavogianni T, Adami ME, Damoraki G, Koufargyris P, Karageorgos A, Bolanou A, Koenen H, van Crevel R, Droggiti DI, Renieris G, Papadopoulos A, Netea MG (2020). Activate: randomized clinical trial of bcg vaccination against infection in the elderly. Cell.

[bib66] Gomez Perdiguero E, Klapproth K, Schulz C, Busch K, Azzoni E, Crozet L, Garner H, Trouillet C, de Bruijn MF, Geissmann F, Rodewald H-R (2015). Tissue-resident macrophages originate from yolk-sac-derived erythro-myeloid progenitors. Nature.

[bib67] Goodridge HS, Ahmed SS, Curtis N, Kollmann TR, Levy O, Netea MG, Pollard AJ, van Crevel R, Wilson CB (2016). Harnessing the beneficial heterologous effects of vaccination. Nature Reviews. Immunology.

[bib68] Guan C, Fernandes FC, Franco OL, de la Fuente-Nunez C (2025). Leveraging large language models for peptide antibiotic design. Cell Reports. Physical Science.

[bib69] Guenther C, Faisal I, Fusciello M, Sokolova M, Harjunpää H, Ilander M, Tallberg R, Vartiainen MK, Alon R, Gonzalez-Granado JM, Cerullo V, Fagerholm SC (2021). β2-integrin adhesion regulates dendritic cell epigenetic and transcriptional landscapes to restrict dendritic cell maturation and tumor rejection. Cancer Immunology Research.

[bib70] Hackett T-L, Vriesde NRTF, Al-Fouadi M, Mostaco-Guidolin L, Maftoun D, Hsieh A, Coxson N, Usman K, Sin DD, Booth S, Osei ET (2022). The role of the dynamic lung extracellular matrix environment on fibroblast morphology and inflammation. Cells.

[bib71] Harjunpää H, Somermäki R, Saldo Rubio G, Fusciello M, Feola S, Faisal I, Nieminen AI, Wang L, Llort Asens M, Zhao H, Eriksson O, Cerullo V, Fagerholm SC (2024). Loss of β2-integrin function results in metabolic reprogramming of dendritic cells, leading to increased dendritic cell functionality and anti-tumor responses. Oncoimmunology.

[bib72] Hecker M, Mülsch A, Bassenge E, Förstermann U, Busse R (1994). Subcellular localization and characterization of nitric oxide synthase(s) in endothelial cells: physiological implications. The Biochemical Journal.

[bib73] Heng Y, Zhang X, Borggrewe M, van Weering HRJ, Brummer ML, Nijboer TW, Joosten LAB, Netea MG, Boddeke EWGM, Laman JD, Eggen BJL (2021). Systemic administration of β-glucan induces immune training in microglia. Journal of Neuroinflammation.

[bib74] Heo S-J, Han WM, Szczesny SE, Cosgrove BD, Elliott DM, Lee DA, Duncan RL, Mauck RL (2016). Mechanically induced chromatin condensation requires cellular contractility in mesenchymal stem cells. Biophysical Journal.

[bib75] Higgins JPT, Soares-Weiser K, López-López JA, Kakourou A, Chaplin K, Christensen H, Martin NK, Sterne JAC, Reingold AL (2016). Association of BCG, DTP, and measles containing vaccines with childhood mortality: systematic review. BMJ.

[bib76] Hoagland H (1944). Adventures in biological engineering. Science.

[bib77] Hong M, Sandalova E, Low D, Gehring AJ, Fieni S, Amadei B, Urbani S, Chong Y-S, Guccione E, Bertoletti A (2015). Trained immunity in newborn infants of HBV-infected mothers. Nature Communications.

[bib78] Hood JD, Cheresh DA (2002). Role of integrins in cell invasion and migration. Nature Reviews. Cancer.

[bib79] Hotz C, Wagenaar TR, Gieseke F, Bangari DS, Callahan M, Cao H, Diekmann J, Diken M, Grunwitz C, Hebert A, Hsu K, Bernardo M, Karikó K, Kreiter S, Kuhn AN, Levit M, Malkova N, Masciari S, Pollard J, Qu H, Ryan S, Selmi A, Schlereth J, Singh K, Sun F, Tillmann B, Tolstykh T, Weber W, Wicke L, Witzel S, Yu Q, Zhang YA, Zheng G, Lager J, Nabel GJ, Sahin U, Wiederschain D (2021). Local delivery of mRNA-encoded cytokines promotes antitumor immunity and tumor eradication across multiple preclinical tumor models. Science Translational Medicine.

[bib80] Hua S, Marks E, Schneider JJ, Keely S (2015). Advances in oral nano-delivery systems for colon targeted drug delivery in inflammatory bowel disease: selective targeting to diseased versus healthy tissue. Nanomedicine.

[bib81] Ideker T, Winslow LR, Lauffenburger DA (2006). Bioengineering and Systems Biology. Annals of Biomedical Engineering.

[bib82] Inmunotek SL (2024). A Phase I/II Randomized, Prospective, Double-Blind, Placebo-Controlled, Single-Center Study to Evaluate the Ability of Sublingual MV130 to Induce the Expression of Trained Immunity in Peripheral Blood Cells. Clinicaltrials.gov.

[bib83] Institut Pasteur (2024). Description of the Immune Response to Yellow Fever Vaccination. https://clinicaltrials.gov/study/NCT06718127.

[bib84] Jang M, An J, Oh SW, Lim JY, Kim J, Choi JK, Cheong J-H, Kim P (2021). Matrix stiffness epigenetically regulates the oncogenic activation of the Yes-associated protein in gastric cancer. Nature Biomedical Engineering.

[bib85] Jennings VA, Scott GB, Rose AMS, Scott KJ, Migneco G, Keller B, Reilly K, Donnelly O, Peach H, Dewar D, Harrington KJ, Pandha H, Samson A, Vile RG, Melcher AA, Errington-Mais F (2019). Potentiating oncolytic virus-induced immune-mediated tumor cell killing using histone deacetylase inhibition. Molecular Therapy.

[bib86] Jiayuan Z (2020). A Multicenter, Randomized, Controlled Clinical Trial of Tissue-Engineered Skin Grafts With Autologous Scar Dermal Scaffolds for the Repair of Hypertrophic Scars. Clinicaltrials.gov.

[bib87] John SP, Singh A, Sun J, Pierre MJ, Alsalih L, Lipsey C, Traore Z, Balcom-Luker S, Bradfield CJ, Song J, Markowitz TE, Smelkinson M, Ferrer M, Fraser IDC (2022). Small-molecule screening identifies Syk kinase inhibition and rutaecarpine as modulators of macrophage training and SARS-CoV-2 infection. Cell Reports.

[bib88] Jones DL, Hallström GF, Jiang X, Locke RC, Evans MK, Bonnevie ED, Srikumar A, Leahy TP, Nijsure MP, Boerckel JD, Mauck RL, Dyment NA (2023). Mechanoepigenetic regulation of extracellular matrix homeostasis via Yap and Taz. PNAS.

[bib89] Kalafati L, Kourtzelis I, Schulte-Schrepping J, Li X, Hatzioannou A, Grinenko T, Hagag E, Sinha A, Has C, Dietz S, de Jesus Domingues AM, Nati M, Sormendi S, Neuwirth A, Chatzigeorgiou A, Ziogas A, Lesche M, Dahl A, Henry I, Subramanian P, Wielockx B, Murray P, Mirtschink P, Chung KJ, Schultze JL, Netea MG, Hajishengallis G, Verginis P, Mitroulis I, Chavakis T (2020). Innate immune training of granulopoiesis promotes anti-tumor activity. Cell.

[bib90] Kalkal A, Pradhan R, Kadian S, Manik G, Packirisamy G (2020). Biofunctionalized graphene quantum dots based fluorescent biosensor toward efficient detection of small cell lung cancer. ACS Applied Bio Materials.

[bib91] Kaufman J, Graf BA, Leung EC, Pollock SJ, Koumas L, Reddy SY, Blieden TM, Smith TJ, Phipps RP (2001). Fibroblasts as sentinel cells: role of the CDcd40-CDcd40 ligand system in fibroblast activation and lung inflammation and fibrosis. Chest.

[bib92] Kaufman HL, Shalhout SZ, Iodice G (2022). Talimogene laherparepvec: moving from first-in-class to best-in-class. Frontiers in Molecular Biosciences.

[bib93] Kaufmann E, Sanz J, Dunn JL, Khan N, Mendonça LE, Pacis A, Tzelepis F, Pernet E, Dumaine A, Grenier J-C, Mailhot-Léonard F, Ahmed E, Belle J, Besla R, Mazer B, King IL, Nijnik A, Robbins CS, Barreiro LB, Divangahi M (2018). BCG educates hematopoietic stem cells to generate protective innate immunity against tuberculosis. Cell.

[bib94] Kim JY, Choi WI, Kim YH, Tae G (2013). Brain-targeted delivery of protein using chitosan- and RVG peptide-conjugated, pluronic-based nano-carrier. Biomaterials.

[bib95] Kim NG, Gumbiner BM (2015). Adhesion to fibronectin regulates Hippo signaling via the FAK-Src-PI3K pathway. The Journal of Cell Biology.

[bib96] Kleinnijenhuis J, Quintin J, Preijers F, Joosten LAB, Ifrim DC, Saeed S, Jacobs C, van Loenhout J, de Jong D, Stunnenberg HG, Xavier RJ, van der Meer JWM, van Crevel R, Netea MG (2012). Bacille Calmette-Guerin induces NOD2-dependent nonspecific protection from reinfection via epigenetic reprogramming of monocytes. PNAS.

[bib97] Knight HR, Ketter E, Ung T, Weiss A, Ajit J, Chen Q, Shen J, Ip KM, Chiang C-Y, Barreiro L, Esser-Kahn A (2024). High-throughput screen identifies non inflammatory small molecule inducers of trained immunity. PNAS.

[bib98] Koelwyn GJ, Newman AAC, Afonso MS, van Solingen C, Corr EM, Brown EJ, Albers KB, Yamaguchi N, Narke D, Schlegel M, Sharma M, Shanley LC, Barrett TJ, Rahman K, Mezzano V, Fisher EA, Park DS, Newman JD, Quail DF, Nelson ER, Caan BJ, Jones LW, Moore KJ (2020). Myocardial infarction accelerates breast cancer via innate immune reprogramming. Nature Medicine.

[bib99] Kumar M, Kulkarni P, Liu S, Chemuturi N, Shah DK (2023). Nanoparticle biodistribution coefficients: a quantitative approach for understanding the tissue distribution of nanoparticles. Advanced Drug Delivery Reviews.

[bib100] Larsen SB, Cowley CJ, Sajjath SM, Barrows D, Yang Y, Carroll TS, Fuchs E (2021). Establishment, maintenance, and recall of inflammatory memory. Cell Stem Cell.

[bib101] Larson RC, Maus MV (2021). Recent advances and discoveries in the mechanisms and functions of CAR T cells. Nature Reviews. Cancer.

[bib102] Lee A, Scott MKD, Wimmers F, Arunachalam PS, Luo W, Fox CB, Tomai M, Khatri P, Pulendran B (2022). A molecular atlas of innate immunity to adjuvanted and live attenuated vaccines, in mice. Nature Communications.

[bib103] Lee G, Ahn H, Lee E, Lee GS (2023a). The role of NLRP3 inflammasomes in trained immunity. Frontiers in Bioscience.

[bib104] Lee YH, Medhi H, Liu X, Ha IH, Nam KT, Ploegh H (2023b). Selective targeting of nanobody-modified gold nanoparticles to distinct cell types. ACS Applied Materials & Interfaces.

[bib105] Lee H, Cho HJ, Han Y, Lee SH (2024). Mid- to long-term efficacy and safety of stem cell therapy for acute myocardial infarction: a systematic review and meta-analysis. Stem Cell Research & Therapy.

[bib106] Leonard A, Tisdale JF (2024). A new frontier: FDA approvals for gene therapy in sickle cell disease. Molecular Therapy.

[bib107] Li Z, Han P, You Z-H, Li X, Zhang Y, Yu H, Nie R, Chen X (2017). In silico prediction of drug-target interaction networks based on drug chemical structure and protein sequences. Scientific Reports.

[bib108] Li J, Jiang X, Li H, Gelinsky M, Gu Z (2021). Tailoring materials for modulation of macrophage fate. Advanced Materials.

[bib109] Li X, Wang H, Yu X, Saha G, Kalafati L, Ioannidis C, Mitroulis I, Netea MG, Chavakis T, Hajishengallis G (2022). Maladaptive innate immune training of myelopoiesis links inflammatory comorbidities. Cell.

[bib110] Li Q, Wang R, Zhang Z, Wang H, Lu X, Zhang J, Kong AP-S, Tian XY, Chan H-F, Chung AC-K, Cheng JC-Y, Jiang Q, Lee WY-W (2023a). Sirt3 mediates the benefits of exercise on bone in aged mice. Cell Death and Differentiation.

[bib111] Li T, Yang Y, Qi H, Cui W, Zhang L, Fu X, He X, Liu M, Li P-F, Yu T (2023b). CRISPR/Cas9 therapeutics: progress and prospects. Signal Transduction and Targeted Therapy.

[bib112] Li J, Zhu J, Gray O, Sobreira DR, Wu D, Huang R-T, Miao B, Sakabe NJ, Krause MD, Kaikkonen MU, Romanoski CE, Nobrega MA, Fang Y (2024). Mechanosensitive super-enhancers regulate genes linked to atherosclerosis in endothelial cells. The Journal of Cell Biology.

[bib113] Liao J, Wang Q, Wu F, Huang Z (2022). In silico methods for identification of potential active sites of therapeutic targets. Molecules.

[bib114] Libmeldy (2020). European Medicines Agency. https://www.ema.europa.eu/en/medicines/human/EPAR/libmeldy.

[bib115] Liu M, Su Y, Chen M, Wang J, Liu M, Dai Y, Wang C, Luo X, Lai C, Liu M, Ding J, Li C, Hu Y, Tang X, Liu X, Deng Y, Song Y (2022). A preliminary study of the innate immune memory of Kupffer cells induced by PEGylated nanoemulsions. Journal of Controlled Release.

[bib116] Liu J, Liu J, Mu W, Ma Q, Zhai X, Jin B, Liu Y, Zhang N (2024a). Delivery strategy to enhance the therapeutic efficacy of liver fibrosis via nanoparticle drug delivery systems. ACS Nano.

[bib117] Liu G, Ma N, Cheng K, Feng Q, Ma X, Yue Y, Li Y, Zhang T, Gao X, Liang J, Zhang L, Wang X, Ren Z, Fu Y-X, Zhao X, Nie G (2024b). Bacteria-derived nanovesicles enhance tumour vaccination by trained immunity. Nature Nanotechnology.

[bib118] Lu H, Zhang S, Wang J, Chen Q (2021). A review on polymer and lipid-based nanocarriers and its application to nano-pharmaceutical and food-based systems. Frontiers in Nutrition.

[bib119] Luo J, Liu X, Li J, Chen Q, Chen J (2024). Controllable Protein Design by Prefix-Tuning Protein Language Models. bioRxiv.

[bib120] Ma C, Wu M, Ye W, Huang Z, Ma X, Wang W, Wang W, Huang Y, Pan X, Wu C (2021). Inhalable solid lipid nanoparticles for intracellular tuberculosis infection therapy: macrophage-targeting and pH-sensitive properties. Drug Delivery and Translational Research.

[bib121] Ma Y (2025). Amplification of Autologous Epidermal Cells to Repair Large Area Deep Wounds. Clinicaltrials.gov.

[bib122] Madani A, Krause B, Greene ER, Subramanian S, Mohr BP, Holton JM, Olmos JL, Xiong C, Sun ZZ, Socher R, Fraser JS, Naik N (2023). Large language models generate functional protein sequences across diverse families. Nature Biotechnology.

[bib123] Mallick I, Panchal P, Kadam S, Mohite P, Scheele J, Seiz W, Agarwal A, Sharma OP (2023). In-silico identification and prioritization of therapeutic targets of asthma. Scientific Reports.

[bib124] Mills TS, Kain B, Burchill MA, Danis E, Lucas ED, Culp-Hill R, Cowan CM, Schleicher WE, Patel SB, Tran BT, Cao R, Goodspeed A, Ferrara S, Bevers S, Jirón Tamburini BA, Roede JR, D’Alessandro A, King KY, Pietras EM (2024). A distinct metabolic and epigenetic state drives trained immunity in HSC-derived macrophages from autoimmune mice. Cell Stem Cell.

[bib125] Minocha N, Kumar V (2022). Nanostructure system: liposome – a bioactive carrier in drug delivery systems. Materials Today.

[bib126] Miranti CK, Brugge JS (2002). Sensing the environment: a historical perspective on integrin signal transduction. Nature Cell Biology.

[bib127] Mitragotri S, Lahann J (2009). Physical approaches to biomaterial design. Nature Materials.

[bib128] Mitroulis I, Ruppova K, Wang B, Chen LS, Grzybek M, Grinenko T, Eugster A, Troullinaki M, Palladini A, Kourtzelis I, Chatzigeorgiou A, Schlitzer A, Beyer M, Joosten LAB, Isermann B, Lesche M, Petzold A, Simons K, Henry I, Dahl A, Schultze JL, Wielockx B, Zamboni N, Mirtschink P, Coskun Ü, Hajishengallis G, Netea MG, Chavakis T (2018). Modulation of myelopoiesis progenitors is an integral component of trained immunity. Cell.

[bib129] Moorlag SJCFM, Khan N, Novakovic B, Kaufmann E, Jansen T, van Crevel R, Divangahi M, Netea MG (2020). β-glucan induces protective trained immunity against *Mycobacterium tuberculosis* infection: a key role for IL-1. Cell Reports.

[bib130] Moorlag SJCFM, Folkman L, Ter Horst R, Krausgruber T, Barreca D, Schuster LC, Fife V, Matzaraki V, Li W, Reichl S, Mourits VP, Koeken VACM, de Bree LCJ, Dijkstra H, Lemmers H, van Cranenbroek B, van Rijssen E, Koenen HJPM, Joosten I, Xu C-J, Li Y, Joosten LAB, van Crevel R, Netea MG, Bock C (2024). Multi-omics analysis of innate and adaptive responses to BCG vaccination reveals epigenetic cell states that predict trained immunity. Immunity.

[bib131] Mulder WJM, Ochando J, Joosten LAB, Fayad ZA, Netea MG (2019). Therapeutic targeting of trained immunity. Nature Reviews. Drug Discovery.

[bib132] Naik S, Larsen SB, Gomez NC, Alaverdyan K, Sendoel A, Yuan S, Polak L, Kulukian A, Chai S, Fuchs E (2017). Inflammatory memory sensitizes skin epithelial stem cells to tissue damage. Nature.

[bib133] Naik S, Fuchs E (2022). Inflammatory memory and tissue adaptation in sickness and in health. Nature.

[bib134] Nardone G, Oliver-De La Cruz J, Vrbsky J, Martini C, Pribyl J, Skládal P, Pešl M, Caluori G, Pagliari S, Martino F, Maceckova Z, Hajduch M, Sanz-Garcia A, Pugno NM, Stokin GB, Forte G (2017). YAP regulates cell mechanics by controlling focal adhesion assembly. Nature Communications.

[bib135] Nature (2023). Reflections on bioengineering’s disruptiveness. Nature Reviews Bioengineering.

[bib136] Nava MM, Miroshnikova YA, Biggs LC, Whitefield DB, Metge F, Boucas J, Vihinen H, Jokitalo E, Li X, García Arcos JM, Hoffmann B, Merkel R, Niessen CM, Dahl KN, Wickström SA (2020). Heterochromatin-driven nuclear softening protects the genome against mechanical stress-induced damage. Cell.

[bib137] Nerem R (1997). The Emergence of Bioengineering. National Academy of Engineering Website. https://www.nae.edu/7543/TheEmergenceofBioengineering.

[bib138] Netea MG, Quintin J, van der Meer JWM (2011). Trained immunity: a memory for innate host defense. Cell Host & Microbe.

[bib139] Netea MG, Joosten LAB, Latz E, Mills KHG, Natoli G, Stunnenberg HG, O’Neill LAJ, Xavier RJ (2016). Trained immunity: a program of innate immune memory in health and disease. Science.

[bib140] Netea MG, Joosten LAB (2018). Trained immunity and local innate immune memory in the lung. Cell.

[bib141] Netea MG, Domínguez-Andrés J, Barreiro LB, Chavakis T, Divangahi M, Fuchs E, Joosten LAB, van der Meer JWM, Mhlanga MM, Mulder WJM, Riksen NP, Schlitzer A, Schultze JL, Stabell Benn C, Sun JC, Xavier RJ, Latz E (2020). Defining trained immunity and its role in health and disease. Nature Reviews. Immunology.

[bib142] Ni Y, Qi H, Zhang F, Jiang S, Tang Q, Cai W, Mo W, Miron RJ, Zhang Y (2023). Macrophages modulate stiffness-related foreign body responses through plasma membrane deformation. PNAS.

[bib143] Niazi SK, Mariam Z (2023). Computer-aided drug design and drug discovery: a prospective analysis. Pharmaceuticals.

[bib144] Novakovic B, Habibi E, Wang S-Y, Arts RJW, Davar R, Megchelenbrink W, Kim B, Kuznetsova T, Kox M, Zwaag J, Matarese F, van Heeringen SJ, Janssen-Megens EM, Sharifi N, Wang C, Keramati F, Schoonenberg V, Flicek P, Clarke L, Pickkers P, Heath S, Gut I, Netea MG, Martens JHA, Logie C, Stunnenberg HG (2016). β-glucan reverses the epigenetic state of LPS-Induced Immunological Tolerance. Cell.

[bib145] Novakovic B, Stunnenberg HG (2017). I remember you: epigenetic priming in epithelial stem cells. Immunity.

[bib146] Ochando J, Mulder WJM, Madsen JC, Netea MG, Duivenvoorden R (2023). Trained immunity - basic concepts and contributions to immunopathology. Nature Reviews. Nephrology.

[bib147] O’Farrell A, Niu Z, Li J, Eyndhoven LC, Sarma K, Raj A (2025). Innate Immune Memory Is Stimulus Specific. bioRxiv.

[bib148] Owens BMJ, Simmons A (2013). Intestinal stromal cells in mucosal immunity and homeostasis. Mucosal Immunology.

[bib149] Owens BMJ, Steevels TAM, Dudek M (2013). CD90(+) stromal cells are non-professional innate immune effectors of the human colonic mucosa. Frontiers in Immunology.

[bib150] Owens BMJ (2015). Inflammation, innate immunity, and the intestinal stromal cell niche: opportunities and challenges. Frontiers in Immunology.

[bib151] Peng J (2024). Alginate-gelatin hydrogel promotes the neurogenic differentiation potential of bone marrow CD117^+^ hematopoietic stem cells. Regenerative Therapy.

[bib152] Poletti V, Mavilio F (2021). Designing lentiviral vectors for gene therapy of genetic diseases. Viruses.

[bib153] Priem B, van Leent MMT, Teunissen AJP, Sofias AM, Mourits VP, Willemsen L, Klein ED, Oosterwijk RS, Meerwaldt AE, Munitz J, Prévot G, Vera Verschuur A, Nauta SA, van Leeuwen EM, Fisher EL, de Jong KAM, Zhao Y, Toner YC, Soultanidis G, Calcagno C, Bomans PHH, Friedrich H, Sommerdijk N, Reiner T, Duivenvoorden R, Zupančič E, Di Martino JS, Kluza E, Rashidian M, Ploegh HL, Dijkhuizen RM, Hak S, Pérez-Medina C, Bravo-Cordero JJ, de Winther MPJ, Joosten LAB, van Elsas A, Fayad ZA, Rialdi A, Torre D, Guccione E, Ochando J, Netea MG, Griffioen AW, Mulder WJM (2020). Trained immunity-promoting nanobiologic therapy suppresses tumor growth and potentiates checkpoint inhibition. Cell.

[bib154] Pulido JIR (2024). Evaluation of Clinical and Radiographic Parameters in Bony Defects Treated With Recombinant Human Platelet-Derived Growth Factor in Combination With Allograft. Clinicaltrials.Gov.

[bib155] Quanta Medical (2025). First-in-Human Study of MATTISSE Tissue Engineering Chamber in Adult Female After Total Mastectomy for Breast Cancer in Immediate or Delayed 2-Stage Tissue Expander Reconstruction or Conversion from Implant-Based to Autologous Reconstruction. Clinicaltrials.Gov.

[bib156] Radboud University Medical Center (2023). Trained Immunity of Myeloid Cells and Their 1270 Progenitors in Patients With Non-Medullary Thyroid Carcinoma and Colon Carcinoma. Clinicaltrials.Gov.

[bib157] Radboud University Medical Center (2024). HEPHESTOS - Hereditary Pheochromocytoma Assessment of Tumour Immunologies. Clinicaltrials.Gov.

[bib158] Ramey-Ward AN, Dong Y, Yang J, Ogasawara H, Bremer-Sai EC, Brazhkina O, Franck C, Davis M, Salaita K (2023). Optomechanically actuated hydrogel platform for cell stimulation with spatial and temporal resolution. ACS Biomaterials Science & Engineering.

[bib159] Reiss KA, Angelos MG, Dees EC, Yuan Y, Ueno NT, Pohlmann PR, Johnson ML, Chao J, Shestova O, Serody JS, Schmierer M, Kremp M, Ball M, Qureshi R, Schott BH, Sonawane P, DeLong SC, Christiano M, Swaby RF, Abramson S, Locke K, Barton D, Kennedy E, Gill S, Cushing D, Klichinsky M, Condamine T, Abdou Y (2025). CAR-macrophage therapy for HER2-overexpressing advanced solid tumors: a phase 1 trial. Nature Medicine.

[bib160] Riksen NP, Netea MG (2021). Immunometabolic control of trained immunity. Molecular Aspects of Medicine.

[bib161] Rojas LA, Sethna Z, Soares KC, Olcese C, Pang N, Patterson E, Lihm J, Ceglia N, Guasp P, Chu A, Yu R, Chandra AK, Waters T, Ruan J, Amisaki M, Zebboudj A, Odgerel Z, Payne G, Derhovanessian E, Müller F, Rhee I, Yadav M, Dobrin A, Sadelain M, Łuksza M, Cohen N, Tang L, Basturk O, Gönen M, Katz S, Do RK, Epstein AS, Momtaz P, Park W, Sugarman R, Varghese AM, Won E, Desai A, Wei AC, D’Angelica MI, Kingham TP, Mellman I, Merghoub T, Wolchok JD, Sahin U, Türeci Ö, Greenbaum BD, Jarnagin WR, Drebin J, O’Reilly EM, Balachandran VP (2023). Personalized RNA neoantigen vaccines stimulate T cells in pancreatic cancer. Nature.

[bib162] Rosenblum D, Naik S (2022). Epithelial-immune crosstalk in health and disease. Current Opinion in Genetics & Development.

[bib163] Russell SK, Harrison JK, Olson BS, Lee HJ, O’Brien VP, Xing X, Livny J, Yu L, Roberson EDO, Bomjan R, Fan C, Sha M, Estfanous S, Amer AO, Colonna M, Stappenbeck TS, Wang T, Hannan TJ, Hultgren SJ (2023). Uropathogenic *Escherichia coli* infection-induced epithelial trained immunity impacts urinary tract disease outcome. Nature Microbiology.

[bib164] Saeed S, Quintin J, Kerstens HHD, Rao NA, Aghajanirefah A, Matarese F, Cheng S-C, Ratter J, Berentsen K, van der Ent MA, Sharifi N, Janssen-Megens EM, Ter Huurne M, Mandoli A, van Schaik T, Ng A, Burden F, Downes K, Frontini M, Kumar V, Giamarellos-Bourboulis EJ, Ouwehand WH, van der Meer JWM, Joosten LAB, Wijmenga C, Martens JHA, Xavier RJ, Logie C, Netea MG, Stunnenberg HG (2014). Epigenetic programming of monocyte-to-macrophage differentiation and trained innate immunity. Science.

[bib165] Safari H, Kelley WJ, Saito E, Kaczorowski N, Carethers L, Shea LD, Eniola-Adefeso O (2020). Neutrophils preferentially phagocytose elongated particles-An opportunity for selective targeting in acute inflammatory diseases. Science Advances.

[bib166] Sahin U, Oehm P, Derhovanessian E, Jabulowsky RA, Vormehr M, Gold M, Maurus D, Schwarck-Kokarakis D, Kuhn AN, Omokoko T, Kranz LM, Diken M, Kreiter S, Haas H, Attig S, Rae R, Cuk K, Kemmer-Brück A, Breitkreuz A, Tolliver C, Caspar J, Quinkhardt J, Hebich L, Stein M, Hohberger A, Vogler I, Liebig I, Renken S, Sikorski J, Leierer M, Müller V, Mitzel-Rink H, Miederer M, Huber C, Grabbe S, Utikal J, Pinter A, Kaufmann R, Hassel JC, Loquai C, Türeci Ö (2020). An RNA vaccine drives immunity in checkpoint-inhibitor-treated melanoma. Nature.

[bib167] Schaefer TM, Desouza K, Fahey JV, Beagley KW, Wira CR (2004). Toll-like receptor (TLR) expression and TLR-mediated cytokine/chemokine production by human uterine epithelial cells. Immunology.

[bib168] Schrijver DP, Röring RJ, Deckers J, de Dreu A, Toner YC, Prevot G, Priem B, Munitz J, Nugraha EG, van Elsas Y, Azzun A, Anbergen T, Groh LA, Becker AMD, Pérez-Medina C, Oosterwijk RS, Novakovic B, Moorlag SJCFM, Jansen A, Pickkers P, Kox M, Beldman TJ, Kluza E, van Leent MMT, Teunissen AJP, van der Meel R, Fayad ZA, Joosten LAB, Fisher EA, Merkx M, Netea MG, Mulder WJM (2023). Resolving sepsis-induced immunoparalysis via trained immunity by targeting interleukin-4 to myeloid cells. Nature Biomedical Engineering.

[bib169] Scott AK, Casas E, Schneider SE, Swearingen AR, Van Den Elzen CL, Seelbinder B, Barthold JE, Kugel JF, Stern JL, Foster KJ, Emery NC, Brumbaugh J, Neu CP (2023). Mechanical memory stored through epigenetic remodeling reduces cell therapeutic potential. Biophysical Journal.

[bib170] Shi S, Zhong H, Zhang Y, Mei Q (2024). Targeted delivery of nano-radiosensitizers for tumor radiotherapy. Coordination Chemistry Reviews.

[bib171] Slezak AJ, Mansurov A, Raczy MM, Chang K, Alpar AT, Lauterbach AL, Wallace RP, Weathered RK, Medellin JEG, Battistella C, Gray LT, Marchell TM, Gomes S, Swartz MA, Hubbell JA (2022). Tumor cell-surface binding of immune stimulating polymeric glyco-adjuvant via cysteine-reactive pyridyl disulfide promotes antitumor immunity. ACS Central Science.

[bib172] Soltani L, Varmira K, Nazari M (2024). Comparison of the differentiation of ovine fetal bone-marrow mesenchymal stem cells towards osteocytes on chitosan/alginate/CuO-NPs and chitosan/alginate/FeO-NPs scaffolds. Scientific Reports.

[bib173] Song Y, Soto J, Wong SY, Wu Y, Hoffman T, Akhtar N, Norris S, Chu J, Park H, Kelkhoff DO, Ang CE, Wernig M, Kasko A, Downing TL, Poo M-M, Li S (2024). Biphasic regulation of epigenetic state by matrix stiffness during cell reprogramming. Science Advances.

[bib174] Souto EB, Fangueiro JF, Fernandes AR, Cano A, Sanchez-Lopez E, Garcia ML, Severino P, Paganelli MO, Chaud MV, Silva AM (2022). Physicochemical and biopharmaceutical aspects influencing skin permeation and role of SLN and NLC for skin drug delivery. Heliyon.

[bib175] Su K, Shi L, Sheng T, Yan X, Lin L, Meng C, Wu S, Chen Y, Zhang Y, Wang C, Wang Z, Qiu J, Zhao J, Xu T, Ping Y, Gu Z, Liu S (2024). Reformulating lipid nanoparticles for organ-targeted mRNA accumulation and translation. Nature Communications.

[bib176] Subudhi I, Konieczny P, Prystupa A, Castillo RL, Sze-Tu E, Xing Y, Rosenblum D, Reznikov I, Sidhu I, Loomis C, Lu CP, Anandasabapathy N, Suárez-Fariñas M, Gudjonsson JE, Tsirigos A, Scher JU, Naik S (2024). Metabolic coordination between skin epithelium and type 17 immunity sustains chronic skin inflammation. Immunity.

[bib177] Sun J, Chen J, Mohagheghian E, Wang N (2020). Force-induced gene up-regulation does not follow the weak power law but depends on H3K9 demethylation. Science Advances.

[bib178] Tajik A, Zhang Y, Wei F (2016). Transcription upregulation via force-induced direct stretching of chromatin. Nature Materials.

[bib179] Tang Y, Kim JY, Ip CKM, Bahmani A, Chen Q, Rosenberger MG, Esser-Kahn AP, Ferguson AL (2023). Data-driven discovery of innate immunomodulators *via* machine learning-guided high throughput screening. Chemical Science.

[bib180] Tarancón R, Domínguez-Andrés J, Uranga S, Ferreira AV, Groh LA, Domenech M, González-Camacho F, Riksen NP, Aguilo N, Yuste J, Martín C, Netea MG (2020). New live attenuated tuberculosis vaccine MTBVAC induces trained immunity and confers protection against experimental lethal pneumonia. PLOS Pathogens.

[bib181] Thaxton CS, Rink JS, Naha PC, Cormode DP (2016). Lipoproteins and lipoprotein mimetics for imaging and drug delivery. Advanced Drug Delivery Reviews.

[bib182] Therapeutics SJ (2024). A Phase I Study on Engineering Tumor Infiltrating Lymphocytes Injection (GC203 TIL) for the Treatment of Advanced Malignant Solid Tumors. https://clinicaltrials.gov/study/NCT06375187.

[bib183] Tian H, Zhang T, Qin S, Huang Z, Zhou L, Shi J, Nice EC, Xie N, Huang C, Shen Z (2022). Enhancing the therapeutic efficacy of nanoparticles for cancer treatment using versatile targeted strategies. Journal of Hematology & Oncology.

[bib184] Tong W, Hui H, Shang W, Zhang Y, Tian F, Ma Q, Yang X, Tian J, Chen Y (2021). Highly sensitive magnetic particle imaging of vulnerable atherosclerotic plaque with active myeloperoxidase-targeted nanoparticles. Theranostics.

[bib185] Too NSH, Ho NCW, Adine C, Iyer NG, Fong ELS (2021). Hot or cold: bioengineering immune contextures into in vitro patient-derived tumor models. Advanced Drug Delivery Reviews.

[bib186] Tremblay F, Xiong Q, Shah SS, Ko C-W, Kelly K, Morrison MS, Giancarlo C, Ramirez RN, Hildebrand EM, Voytek SB, El Sebae GK, Wright SH, Lofgren L, Clarkson S, Waters C, Linder SJ, Liu S, Eom T, Parikh S, Weber Y, Martinez S, Malyala P, Abubucker S, Friedland AE, Maeder ML, Lombardo A, Myer VE, Jaffe AB (2025). A potent epigenetic editor targeting human PCSK9 for durable reduction of low-density lipoprotein cholesterol levels. Nature Medicine.

[bib187] Uhl P, Sauter M, Hertlein T, Witzigmann D, Laffleur F, Hofhaus G, Fidelj V, Tursch A, Özbek S, Hopke E, Haberkorn U, Bernkop‐Schnürch A, Ohlsen K, Fricker G, Mier W (2021). Overcoming the mucosal barrier: tetraether lipid‐stabilized liposomal nanocarriers decorated with cell‐penetrating peptides enable oral delivery of vancomycin. Advanced Therapeutics.

[bib188] University Hospital, Basel, Switzerland (2025). Treatment of Patellofemoral Osteoarthritis With Nasal Chondrocyte-Based Engineered Cartilage Implantation in a Randomized, Controlled, Multi-Center Phase II Clinical Trial. Clinicaltrials.Gov.

[bib189] van der Heijden CDCC, Groh L, Keating ST, Kaffa C, Noz MP, Kersten S, van Herwaarden AE, Hoischen A, Joosten LAB, Timmers HJLM, Netea MG, Riksen NP (2020). Catecholamines induce trained immunity in monocytes in vitro and in vivo. Circulation Research.

[bib190] Walk J, de Bree LCJ, Graumans W, Stoter R, van Gemert G-J, van de Vegte-Bolmer M, Teelen K, Hermsen CC, Arts RJW, Behet MC, Keramati F, Moorlag SJCFM, Yang ASP, van Crevel R, Aaby P, de Mast Q, van der Ven AJAM, Stabell Benn C, Netea MG, Sauerwein RW (2019). Outcomes of controlled human malaria infection after BCG vaccination. Nature Communications.

[bib191] Wan Z, Dong Q, Liu Y, Zhang X, Zhang P, Lv L, Zhou Y (2022). Programmed biomolecule delivery orchestrate bone tissue regeneration via MSC recruitment and epigenetic modulation. Chemical Engineering Journal.

[bib192] Wang L, Ma C, Wipf P, Liu H, Su W, Xie XQ (2013). Targethunter: an in silico target identification tool for predicting therapeutic potential of small organic molecules based on chemogenomic database. The AAPS Journal.

[bib193] Wang R, Yao Q, Chen W, Gao F, Li P, Wu J, Yu J, Cao H (2021). Stem cell therapy for Crohn’s disease: systematic review and meta-analysis of preclinical and clinical studies. Stem Cell Research & Therapy.

[bib194] Wang R, Cao S, Bashir MEH, Hesser LA, Su Y, Hong SMC, Thompson A, Culleen E, Sabados M, Dylla NP, Campbell E, Bao R, Nonnecke EB, Bevins CL, Wilson DS, Hubbell JA, Nagler CR (2023a). Treatment of peanut allergy and colitis in mice via the intestinal release of butyrate from polymeric micelles. Nature Biomedical Engineering.

[bib195] Wang X, Liu S, Sun Y, Yu X, Lee SM, Cheng Q, Wei T, Gong J, Robinson J, Zhang D, Lian X, Basak P, Siegwart DJ (2023b). Preparation of selective organ-targeting (SORT) lipid nanoparticles (LNPs) using multiple technical methods for tissue-specific mRNA delivery. Nature Protocols.

[bib196] Wang Z, Liu Y, Hu J, You X, Yang J, Zhang Y, Liu Q, Yang D (2024). Tissue-resident trained immunity in hepatocytes protects against septic liver injury in zebrafish. Cell Reports.

[bib197] Waterhouse DN, Tardi PG, Mayer LD, Bally MB (2001). A comparison of liposomal formulations of doxorubicin with drug administered in free form: changing toxicity profiles. Drug Safety.

[bib198] Whitehead AK, Barnett HH, Caldorera-Moore ME, Newman JJ (2018). Poly (ethylene glycol) hydrogel elasticity influences human mesenchymal stem cell behavior. Regenerative Biomaterials.

[bib199] Wimmers F, Donato M, Kuo A, Ashuach T, Gupta S, Li C, Dvorak M, Foecke MH, Chang SE, Hagan T, De Jong SE, Maecker HT, van der Most R, Cheung P, Cortese M, Bosinger SE, Davis M, Rouphael N, Subramaniam S, Yosef N, Utz PJ, Khatri P, Pulendran B (2021). The single-cell epigenomic and transcriptional landscape of immunity to influenza vaccination. Cell.

[bib200] Wozniak MA, Modzelewska K, Kwong L, Keely PJ (2004). Focal adhesion regulation of cell behavior. Biochimica et Biophysica Acta.

[bib201] Xu J, Guan W, Kong Y, Liu F, Zhao Y, Li G, Yang Y (2022). Regulation of macrophage behavior by chitosan scaffolds with different elastic modulus. Coatings.

[bib202] Yamaguchi Y, Kato Y, Edahiro R, Søndergaard JN, Murakami T, Amiya S, Nameki S, Yoshimine Y, Morita T, Takeshima Y, Sakakibara S, Naito Y, Motooka D, Liu Y-C, Shirai Y, Okita Y, Fujimoto J, Hirata H, Takeda Y, Wing JB, Okuzaki D, Okada Y, Kumanogoh A (2022). Consecutive BNT162b2 mRNA vaccination induces short-term epigenetic memory in innate immune cells. JCI Insight.

[bib203] Yang SQ, Zhang LX, Ge YJ, Zhang JW, Hu JX, Shen CY, Lu AP, Hou TJ, Cao DS (2023). In-silico target prediction by ensemble chemogenomic model based on multi-scale information of chemical structures and protein sequences. Journal of Cheminformatics.

[bib204] Yao Y, Jeyanathan M, Haddadi S, Barra NG, Vaseghi-Shanjani M, Damjanovic D, Lai R, Afkhami S, Chen Y, Dvorkin-Gheva A, Robbins CS, Schertzer JD, Xing Z (2018). Induction of autonomous memory alveolar macrophages requires T Cell Help and Is Critical to Trained Immunity. Cell.

[bib205] Zahedi Tehrani T, Irani S, Ardeshirylajimi A, Seyedjafari E (2024). Natural based hydrogels promote chondrogenic differentiation of human mesenchymal stem cells. Frontiers in Bioengineering and Biotechnology.

[bib206] Zhang B, Moorlag SJ, Dominguez-Andres J, Bulut Ö, Kilic G, Liu Z, van Crevel R, Xu C-J, Joosten LA, Netea MG, Li Y (2022). Single-cell RNA sequencing reveals induction of distinct trained-immunity programs in human monocytes. The Journal of Clinical Investigation.

[bib207] Zhang K, Cheng K (2023). Stem cell-derived exosome versus stem cell therapy. Nature Reviews Bioengineering.

[bib208] Zhao X-B, Chen Y-P, Tan M, Zhao L, Zhai Y-Y, Sun Y-L, Gong Y, Feng X-Q, Du J, Fan Y-B (2021). Extracellular matrix stiffness regulates DNA Methylation by PKCα-dependent nuclear transport of DNMT3L. Advanced Healthcare Materials.

[bib209] Zhou Z, Zhu J, Yeh CF (2025). Precision mRNA Nanomedicine for Targeted Vascular Therapies in ARDS and Atherosclerosis. bioRxiv.

[bib210] Zhuang Y, Wu D, Zhou L (2025). Electrospun Biomimetic Periosteum Promotes Diabetic Bone Defect Regeneration through Regulating Macrophage Polarization and Sequential Drug Release.

[bib211] Ziogas A, Netea MG (2022). Trained immunity-related vaccines: innate immune memory and heterologous protection against infections. Trends in Molecular Medicine.

